# Supplementary material for: Genetics of symptom remission in outpatients with COVID-19
Source: Sci Rep. 2021 May 25;11:10847. doi: 10.1038/s41598-021-90365-6 (PMC8149390; doi:10.1038/s41598-021-90365-6)
Supplement: Supplementary file 2 — Supplementary Information 2. [file 41598_2021_90365_MOESM2_ESM.pdf]

Supplementary Table 1. Duration of COVID-19 symptoms by genotype groups

			Duration of symptoms					
			**All**		**Women**		**Men**	
**rs1173773 (chr 5) in the placebo + colchicine arms**								
**Genotype**	**Symptoms remission**	**Start date**	**N (%)**	**Mean ± SD**	**N (%)**	**Mean ± SD**	**N (%)**	**Mean ± SD**
All	Reported	Baseline	1252 (72.8%)	11.8 ± 7.2	684 (69.7%)	12.8 ± 7.2	568 (77.0%)	10.5 ± 6.8
		Onset		17.0 ± 7.9		17.9 ± 8.1		15.9 ± 7.6
	Censored	-	467 (27.2%)	-	297 (30.3%)	-	170 (23.0%)	-
TT	Reported	Baseline	524 (68.1%)	12.4 ± 7.5	284 (66.2%)	13.5 ± 7.6	240 (70.4%)	11.1 ± 7.2
		Onset		17.9 ± 8.5		19.0 ± 8.8		16.7 ± 7.9
	Censored	-	246 (31.9%)	-	145 (33.8%)	-	101 (29.6%)	-
CT	Reported	Baseline	550 (74.9%)	11.3 ± 6.9	299 (70.5%)	12.3 ± 6.9	251 (81.0%)	10.1 ± 6.6
		Onset		16.2 ± 7.4		17.0 ± 7.4		15.3 ± 7.5
	Censored	-	184 (25.1%)	-	125 (29.5%)	-	59 (19.0%)	-
CC	Reported	Baseline	178 (82.8%)	11.5 ± 7.0	101 (78.9%)	12.5 ± 7.2	77 (88.5%)	10.1 ± 6.4
		Onset		16.8 ± 7.4		17.7 ± 7.7		15.6 ± 6.9
	Censored	-	37 (17.2%)	-	27 (21.1%)	-	10 (11.5%)	-
**rs62575331 (chr 9) in the placebo arm**								
**Genotype**	**Symptoms remission**	**Start date**	**N (%)**	**Mean ± SD**	**N (%)**	**Mean ± SD**	**N (%)**	**Mean ± SD**
All	Reported	Baseline	611 (73.3%)	11.8 ± 7.2	331 (72.0%)	12.9 ± 7.2	280 (75.1%)	10.4 ± 7.0
		Onset		16.8 ± 7.6		17.8 ± 7.6		15.6 ± 7.5
	Censored	-	222 (26.7%)	-	129 (28.0%)	-	93 (24.9%)	-
CC	Reported	Baseline	471 (70.9%)	12.2 ± 7.2	263 (70.9%)	13.0 ± 7.1	208 (71.0%)	11.2 ± 7.2
		Onset		17.2 ± 7.7		17.9 ± 7.6		16.4 ± 7.7
	Censored	-	193 (29.1%)	-	108 (29.1%)	-	85 (29.0%)	-
CG	Reported	Baseline	125 (81.7%)	10.2 ± 6.9	61 (75.3%)	12.4 ± 7.3	64 (88.9%)	8.1 ± 5.9
		Onset		15.4 ± 7.3		17.4 ± 7.5		13.4 ± 6.6
	Censored	-	28 (18.3%)	-	20 (24.7%)	-	8 (11.1%)	-
GG	Reported	Baseline	15 (93.8%)	10.9 ± 7.6	7 (87.5%)	13.4 ± 9.9	8 (100.0%)	8.6 ± 4.5
		Onset		15.6 ± 7.8		18.1 ± 10.5		13.4 ± 4.0
	Censored	-	1 (6.3%)	-	1 (12.5%)	-	0 (0.0%)	-
CG+GG	Reported	Baseline	140 (82.8%)	10.3 ± 7.0	68 (76.4%)	12.5 ± 7.5	72 (90.0%)	8.1 ± 5.7
		Onset		15.4 ± 7.3		17.5 ± 7.8		13.4 ± 6.3
	Censored	-	29 (17.2%)	-	21 (23.6%)	-	8 (10.0%)	-

Baseline refers to the COLCORONA study randomisation visit. *Onset* refers to the reported start date of symptoms by the patient; *Reported* refers to patients who reported symptoms remission during the 30-day follow-up period of the study; *Censored* refers to patients who did not report symptoms remission during the 30-day follow-up period of the study. Chr: chromosome; SD: standard deviation.

Supplementary Table 2. Candidate variants in Locus 1 and Locus 2 (9q33.1 and 5p13.3), and Locus 3 (5p13.3). Results are from the GWAS conducted with genetest.

Locus	Region	SNP	Effect allele	Other allele	Effect allele frequency	beta	SE	P value	N
Locus 1	9q33.1	chr9:115549403:A:G	G	A	0.092	0.437	0.092	2.02E-06	851
Locus 1	9q33.1	chr9:115647521:C:G	G	C	0.116	0.452	0.082	2.95E-08	851
Locus 1	9q33.1	chr9:115649501:G:A	A	G	0.115	0.452	0.082	3.55E-08	851
Locus 1	9q33.1	chr9:115652093:G:A	A	G	0.114	0.452	0.082	3.44E-08	851
Locus 1	9q33.1	chr9:115656803:A:G	G	A	0.117	0.421	0.082	3.38E-07	851
Locus 1	9q33.1	chr9:115662708:A:T	T	A	0.120	0.432	0.082	1.50E-07	851
Locus 2	5p13.3	chr5:32726531:G:A	A	G	0.335	0.309	0.059	1.77E-07	851
Locus 2	5p13.3	chr5:32728976:A:T	T	A	0.334	0.314	0.060	1.38E-07	851
Locus 2	5p13.3	chr5:32729276:A:G	G	A	0.334	0.314	0.060	1.36E-07	851
Locus 2	5p13.3	chr5:32738548:G:A	A	G	0.344	0.300	0.058	2.82E-07	851
Locus 2	5p13.3	chr5:32740326:A:G	G	A	0.345	0.302	0.058	2.21E-07	851
Locus 2	5p13.3	chr5:32740437:T:A	A	T	0.345	0.302	0.058	2.21E-07	851
Locus 2	5p13.3	chr5:32740630:G:A	A	G	0.345	0.302	0.058	2.36E-07	851
Locus 2	5p13.3	chr5:32746441:A:G	G	A	0.344	0.266	0.057	2.59E-06	851
Locus 2	5p13.3	chr5:32748531:G:A	A	G	0.306	0.260	0.058	8.12E-06	851
Locus 2	5p13.3	chr5:32748552:G:A	A	G	0.303	0.264	0.059	6.96E-06	851
Locus 2	5p13.3	chr5:32749196:AT:A	A	AT	0.341	0.272	0.057	2.03E-06	851
Locus 2	5p13.3	chr5:32750309:G:T	T	G	0.300	0.258	0.059	1.28E-05	851
Locus 2	5p13.3	chr5:32750877:T:C	C	T	0.341	0.279	0.057	1.17E-06	851
Locus 2	5p13.3	chr5:32763740:G:A	A	G	0.322	0.266	0.058	4.93E-06	851
Locus 2	5p13.3	chr5:32765383:C:A	A	C	0.319	0.263	0.058	6.37E-06	851
Locus 2	5p13.3	chr5:32773208:C:T	T	C	0.388	0.260	0.056	3.30E-06	851
Locus 3	5p13.3	chr5:32726531:G:A	A	G	0.331	0.213	0.043	7.51E-07	1723
Locus 3	5p13.3	chr5:32728976:A:T	T	A	0.329	0.221	0.043	3.33E-07	1723
Locus 3	5p13.3	chr5:32729276:A:G	G	A	0.330	0.221	0.043	3.41E-07	1723
Locus 3	5p13.3	chr5:32738548:G:A	A	G	0.338	0.208	0.042	8.34E-07	1723
Locus 3	5p13.3	chr5:32740326:A:G	G	A	0.339	0.213	0.042	4.36E-07	1723
Locus 3	5p13.3	chr5:32740437:T:A	A	T	0.339	0.215	0.042	3.32E-07	1723
Locus 3	5p13.3	chr5:32740630:G:A	A	G	0.339	0.213	0.042	4.58E-07	1723
Locus 3	5p13.3	chr5:32746441:A:G	G	A	0.338	0.208	0.041	3.40E-07	1723
Locus 3	5p13.3	chr5:32748531:G:A	A	G	0.298	0.204	0.042	1.31E-06	1723

Locus 3	5p13.3	chr5:32748552:G:A	A	G	0.295	0.207	0.043	1.10E-06	1723
Locus 3	5p13.3	chr5:32749196:AT:A	A	AT	0.336	0.212	0.041	2.33E-07	1723
Locus 3	5p13.3	chr5:32749486:G:A	A	G	0.269	0.201	0.044	4.84E-06	1723
Locus 3	5p13.3	chr5:32750309:G:T	T	G	0.291	0.200	0.043	2.94E-06	1723
Locus 3	5p13.3	chr5:32750877:T:C	C	T	0.337	0.223	0.041	4.96E-08	1723
Locus 3	5p13.3	chr5:32758062:CT:C	C	CT	0.271	0.202	0.043	3.49E-06	1723
Locus 3	5p13.3	chr5:32763740:G:A	A	G	0.315	0.220	0.042	1.20E-07	1723
Locus 3	5p13.3	chr5:32765383:C:A	A	C	0.312	0.218	0.042	1.87E-07	1723
Locus 3	5p13.3	chr5:32765991:C:G	G	C	0.273	0.210	0.044	1.42E-06	1723
Locus 3	5p13.3	chr5:32766311:T:C	C	T	0.275	0.212	0.043	1.06E-06	1723
Locus 3	5p13.3	chr5:32766360:A:G	G	A	0.274	0.213	0.043	9.24E-07	1723
Locus 3	5p13.3	chr5:32766716:T:C	C	T	0.274	0.213	0.043	9.30E-07	1723
Locus 3	5p13.3	chr5:32767621:A:G	G	A	0.274	0.213	0.043	9.32E-07	1723
Locus 3	5p13.3	chr5:32767676:T:C	C	T	0.274	0.213	0.043	9.32E-07	1723
Locus 3	5p13.3	chr5:32767822:T:G	G	T	0.274	0.213	0.043	9.22E-07	1723
Locus 3	5p13.3	chr5:32768528:A:G	G	A	0.276	0.210	0.043	1.15E-06	1723
Locus 3	5p13.3	chr5:32769993:G:T	T	G	0.273	0.213	0.044	9.71E-07	1723
Locus 3	5p13.3	chr5:32771273:A:G	G	A	0.275	0.212	0.043	1.08E-06	1723
Locus 3	5p13.3	chr5:32771719:A:G	G	A	0.274	0.213	0.044	9.83E-07	1723
Locus 3	5p13.3	chr5:32771832:A:G	G	A	0.275	0.211	0.043	1.11E-06	1723
Locus 3	5p13.3	chr5:32771937:A:G	G	A	0.274	0.213	0.044	9.47E-07	1723
Locus 3	5p13.3	chr5:32773208:C:T	T	C	0.377	0.190	0.041	3.09E-06	1723

Supplementary Table 3. Results for the statistical (PAINTOR) and functional (ReguloneDB and DSNetwork) prioritization

							PAINTOR		RelomeDB											DSNetwork															
Locus	Region	dbsnp rsid	chr	pos	dbsnp ref	dbsnp alt	ZSCORE	Posterior Prob	probability	ranking	ChIP	DNase	Footprint	Footprint matched	IC matched max	IC max	PWM	PWM matched	QTL	linsight	iwscoring novel	iwscoring known	bayesdel	deepseq sig log2	eigen nc	eigen pc nc	fathmm nc	fitcons nc	funseq	gwava region	gwava tss	gwava unmatched	remm	ranking (na mean)	ranking (na median)
Locus 2	5p13.3	rs817900	chr5	32740432	A	G	5.18	0.13	0.18	7	FALSE	FALSE	FALSE	FALSE	0.00	0.00	FALSE	FALSE	FALSE	0.046	-1.036	-2.025	-0.92	4.116	0.088	-0.163	0.072	0.061	0.750	0.16	0.17	0.07	0.001	21	16
Locus 3	5p13.3	rs10061804	chr5	32748637	G	T	4.84	0.01	0.61	4	TRUE	TRUE	FALSE	FALSE	0.00	0.00	FALSE	FALSE	FALSE	0.051	-1.336	-2.693	-0.92	2.049	-0.444	-0.231	0.086	0.081	0.924	0.33	0.12	0.02	0.000	37	35
Locus 2	5p13.3	rs1173773	chr5	32750983	T	C	4.86	0.05	0.52	5	FALSE	TRUE	TRUE	FALSE	0.00	1.13	TRUE	FALSE	FALSE	0.068	-1.034	-1.283	-0.92	4.265	-0.073	-0.177	0.062	0.081	0.924	0.42	0.27	0.06	0.009	11	8
Locus 2	5p13.3	rs10061804	chr5	32748637	G	T	4.46	0.03	0.61	4	TRUE	TRUE	FALSE	FALSE	0.00	0.00	FALSE	FALSE	FALSE	0.051	-1.336	-2.693	-0.92	2.049	-0.444	-0.231	0.086	0.081	0.924	0.33	0.12	0.02	0.000	37	35
Locus 2	5p13.3	rs10061807	chr5	32748658	G	T	4.50	0.04	0.19	3a	TRUE	TRUE	FALSE	FALSE	0.00	0.64	TRUE	FALSE	FALSE	0.051	-1.084	-2.141	-0.92	3.145	-0.142	-0.186	0.074	0.081	0.924	0.34	0.11	0.02	0.003	19	13
Locus 3	5p13.3	rs6450925	chr5	32750415	G	T	4.67	0.00	0.61	4	TRUE	TRUE	FALSE	FALSE	0.00	0.00	FALSE	FALSE	FALSE	0.050	-2.071	-2.945	-1.01	2.998	-0.536	-0.235	0.038	0.061	0.127	0.36	0.17	0.01	0.003	36	34
Locus 3	5p13.3	rs10061807	chr5	32748658	G	T	4.87	0.01	0.19	3a	TRUE	TRUE	FALSE	FALSE	0.00	0.64	TRUE	FALSE	FALSE	0.051	-1.084	-2.141	-0.92	3.145	-0.142	-0.186	0.074	0.081	0.924	0.34	0.11	0.02	0.003	19	13
Locus 3	5p13.3	rs817900	chr5	32740432	A	G	5.05	0.01	0.18	7	FALSE	FALSE	FALSE	FALSE	0.00	0.00	FALSE	FALSE	FALSE	0.046	-1.036	-2.025	-0.92	4.116	0.088	-0.163	0.072	0.061	0.750	0.16	0.17	0.07	0.001	21	16
Locus 3	5p13.3	rs1173733	chr5	32766097	C	T	4.82	0.01	1.00	5	FALSE	TRUE	FALSE	FALSE	0.00	0.52	TRUE	FALSE	FALSE	0.052	-1.901	-2.960	NaN	2.345	-0.431	-0.232	0.086	0.061	0.127	0.23	0.26	0.16	0.009	20	25
Locus 3	5p13.3	rs1173734	chr5	32766417	T	C	4.88	0.01	0.13	5	FALSE	TRUE	FALSE	FALSE	0.00	0.00	FALSE	FALSE	FALSE	0.051	-1.349	-2.382	-0.92	2.657	-0.447	-0.234	0.079	0.081	0.924	0.25	0.29	0.15	0.000	31	26
Locus 3	5p13.3	rs1173735	chr5	32771379	A	G	4.88	0.01	0.61	4	TRUE	TRUE	FALSE	FALSE	0.00	0.00	FALSE	FALSE	FALSE	0.121	-1.034	-0.627	-0.84	5.566	0.171	-0.084	0.162	0.061	0.312	0.34	0.48	0.37	0.067	8	6
Locus 3	5p13.3	rs1173736	chr5	32771938	A	G	4.87	0.01	0.33	5	FALSE	TRUE	FALSE	FALSE	0.00	1.50	TRUE	FALSE	FALSE	0.052	-0.823	-1.733	-0.92	3.549	0.027	0.418	0.072	0.061	0.924	0.19	0.33	0.15	0.000	25	17
Locus 1	9q33.1	rs72764716	chr9	118414372	G	A	5.52	0.31	0.69	6	FALSE	FALSE	FALSE	FALSE	0.00	1.57	TRUE	FALSE	FALSE	0.045	-0.615	-0.839	-0.84	3.809	0.188	-0.124	0.159	0.061	0.000	0.38	0.39	0.18	0.555	4	4
Locus 1	9q33.1	rs12006166	chr9	118424987	A	T	5.25	0.11	0.18	6	FALSE	FALSE	FALSE	FALSE	0.00	1.50	TRUE	FALSE	FALSE	0.051	-0.128	-0.516	-0.83	5.692	0.371	-0.118	0.190	0.061	0.623	0.38	0.26	0.04	0.435	2	2
Locus 3	5p13.3	rs1698027	chr5	32738654	G	A	4.93	0.00	0.13	5	FALSE	TRUE	FALSE	FALSE	0.00	0.00	FALSE	FALSE	FALSE	0.068	-0.842	-1.664	-0.83	4.184	0.088	-0.157	0.197	0.061	0.750	0.17	0.25	0.21	0.003	18	10
Locus 2	5p13.3	rs3792751	chr5	32773314	C	T	4.65	0.29	0.32	5	FALSE	TRUE	FALSE	FALSE	0.00	0.60	TRUE	FALSE	FALSE	0.086	-1.808	-2.821	-0.98	2.331	-0.240	-0.203	0.044	0.061	0.127	0.25	0.28	0.09	0.000	35	36
Locus 3	5p13.3	rs3792751	chr5	32773314	C	T	4.66	0.33	0.32	5	FALSE	TRUE	FALSE	FALSE	0.00	0.60	TRUE	FALSE	FALSE	0.086	-1.808	-2.821	-0.98	2.331	-0.240	-0.203	0.044	0.061	0.127	0.25	0.28	0.09	0.000	35	36
Locus 3	5p13.3	rs3792752	chr5	32768634	A	G	4.86	0.01	0.61	4	TRUE	TRUE	FALSE	FALSE	0.00	0.00	FALSE	FALSE	FALSE	0.055	1.635	1.280	-0.89	3.205	0.954	4.467	0.126	0.061	1.546	0.52	0.55	0.45	0.126	5	4
Locus 3	5p13.3	rs3811958	chr5	32772043	A	G	4.90	0.01	0.00	5	FALSE	TRUE	FALSE	FALSE	0.00	1.24	TRUE	FALSE	FALSE	0.113	1.371	1.209	-0.67	5.568	0.596	-0.083	0.429	0.061	1.547	0.29	0.35	0.18	0.650	2	2
Locus 3	5p13.3	rs3811960	chr5	32771825	A	T	4.90	0.01	0.13	5	FALSE	TRUE	FALSE	FALSE	0.00	0.00	FALSE	FALSE	FALSE	0.051	-1.434	-2.687	-0.89	1.755	-0.136	0.402	0.134	0.061	0.127	0.2	0.25	0.19	0.002	34	31
Locus 3	5p13.3	rs696833	chr5	32726637	G	A	4.95	0.01	0.82	3a	TRUE	TRUE	FALSE	FALSE	0.00	0.19	TRUE	FALSE	FALSE	0.044	-1.185	-1.755	-0.92	2.406	-0.220	-0.191	0.071	0.057	0.924	0.42	0.29	0.19	0.001	29	23
Locus 3	5p13.3	rs3811964	chr5	32770099	G	T	4.90	0.01	0.13	5	FALSE	TRUE	FALSE	FALSE	0.00	0.00	FALSE	FALSE	FALSE	0.051	-1.048	-1.550	-0.93	2.610	-0.289	0.528	0.082	0.061	0.924	0.42	0.39	0.33	0.000	28	24
Locus 3	5p13.3	rs3811966	chr5	32766822	T	C	4.91	0.01	0.13	5	FALSE	TRUE	FALSE	FALSE	0.00	0.00	FALSE	FALSE	FALSE	0.051	-1.909	-3.039	-0.92	1.080	-0.439	-0.231	0.092	0.061	0.127	0.28	0.25	0.25	0.000	38	38
Locus 3	5p13.3	rs3811968	chr5	32765489	C	T	5.21	0.04	0.13	5	FALSE	TRUE	FALSE	FALSE	0.00	0.00	FALSE	FALSE	FALSE	0.111	-1.204	-2.395	-0.91	1.812	-0.305	-0.210	0.105	0.061	0.924	0.29	0.29	0.09	0.001	33	29
Locus 2	5p13.3	rs696833	chr5	32726637	G	A	5.22	0.16	0.82	3a	TRUE	TRUE	FALSE	FALSE	0.00	0.19	TRUE	FALSE	FALSE	0.044	-1.185	-1.755	-0.92	2.406	-0.220	-0.191	0.071	0.057	0.924	0.42	0.29	0.19	0.001	29	23
Locus 2	5p13.3	rs3811968	chr5	32765489	C	T	4.51	0.03	0.13	5	FALSE	TRUE	FALSE	FALSE	0.00	0.00	FALSE	FALSE	FALSE	0.111	-1.204	-2.395	-0.91	1.812	-0.305	-0.210	0.105	0.061	0.924	0.29	0.29	0.09	0.001	33	29
Locus 2	5p13.3	rs1173730	chr5	32763846	G	A	4.57	0.03	0.13	5	FALSE	TRUE	FALSE	FALSE	0.00	0.00	FALSE	FALSE	FALSE	0.051	-1.318	-2.460	-0.99	2.777	-0.175	-0.196	0.039	0.061	0.750	0.21	0.19	0.06	0.000	30	21
Locus 3	5p13.3	rs1173730	chr5	32763846	G	A	5.29	0.08	0.13	5	FALSE	TRUE	FALSE	FALSE	0.00	0.00	FALSE	FALSE	FALSE	0.051	-1.318	-2.460	-0.99	2.777	-0.175	-0.196	0.039	0.061	0.750	0.21	0.19	0.06	0.000	30	21
Locus 3	5p13.3	rs3828586	chr5	32746547	A	G	5.10	0.01	0.11	6	FALSE	FALSE	TRUE	FALSE	0.00	0.00	FALSE	FALSE	FALSE	0.084	-0.938	-1.551	-0.94	2.980	0.058	-0.154	0.057	0.081	0.924	0.44	0.21	0.06	0.000	16	12
Locus 3	5p13.3	rs1173773	chr5	32750983	T	C	5.45	0.86	0.52	5	FALSE	TRUE	TRUE	FALSE	0.00	1.13	TRUE	FALSE	FALSE	0.068	-1.034	-1.283	-0.92	4.265	-0.073	-0.177	0.062	0.081	0.924	0.42	0.27	0.06	0.009	11	8
Locus 2	5p13.3	rs3828586	chr5	32746547	A	G	4.70	0.03	0.11	6	FALSE	FALSE	TRUE	FALSE	0.00	0.00	FALSE	FALSE	FALSE	0.084	-0.938	-1.551	-0.94	2.980	0.058	-0.154	0.057	0.081	0.924	0.44	0.21	0.06	0.000	16	12
Locus 1	9q33.1	rs62575331	chr9	118409800	C	G	5.54	0.36	0.18	7	FALSE	FALSE	FALSE	FALSE	0.00	0.00	FALSE	FALSE	FALSE	0.505	1.490	1.834	-0.24	6.300	0.959	-0.024	0.961	0.061	0.002	0.37	0.25	0.07	0.716	1	1
Locus 1	9q33.1	rs62575332	chr9	118419082	A	G	5.10	0.08	0.37	6	FALSE	FALSE	FALSE	FALSE	0.00	0.84	TRUE	FALSE	FALSE	0.044	-1.707	-2.647	-0.92	2.696	-0.313	-0.215	0.075	0.061	0.000	0.38	0.09	0.01	0.158	5	5
Locus 1	9q33.1	rs62576696	chr9	118311682	A	G	4.75	0.05	0.30	6	FALSE	FALSE	FALSE	FALSE	0.00	0.30	TRUE	FALSE	FALSE	0.041	-0.875	-2.216	-0.89	2.368	-0.076	-0.167	0.124	0.054	0.185	0.18	0.19	0.11	0.480	6	6
Locus 3	5p13.3	rs817901	chr5	32740543	T	A	5.10	0.01	0.18	7	FALSE	FALSE	FALSE	FALSE	0.00	0.00	FALSE	FALSE	FALSE	0.044	-1.546	-2.923	-0.85	1.403	-0.141	-0.198	0.161	0.061	0.127	0.17	0.14	0.09	0.000	32	28
Locus 2	5p13.3	rs6450925	chr5	32750415	G	T	4.36	0.03	0.61	4	TRUE	TRUE	FALSE	FALSE	0.00	0.00	FALSE	FALSE	FALSE	0.050	-2.071	-2.945	-1.01	2.998	-0.536	-0.235	0.038	0.061	0.127	0.36	0.17	0.01	0.003	36	34
Locus 1	9q33.1	rs72764714	chr9	118411780	G	A	5.51	0.31	0.21	6	FALSE	FALSE	FALSE	FALSE	0.00	0.77	TRUE	FALSE	FALSE	0.050	-0.569	-0.904	-0.84	4.366	0.212	-0.059	0.164	0.061	0.000	0.26	0.45	0.19	0.556	3	3
Locus 3	5p13.3	rs7713015	chr5	32767782	T	C	4.91	0.01	0.13	5	FALSE	TRUE	FALSE	FALSE	0.00	0.00	FALSE	FALSE	FALSE	0.051	-1.799	-3.000	-0.94	1.116	-0.940	-0.268	0.075	0.061	0.924	0.31	0.26	0.16	0.001	39	39
Locus 3	5p13.3	rs7716901	chr5	32767928	T	G	4.91	0.01	0.89	5	TRUE	FALSE	FALSE	FALSE	0.00	0.46	TRUE	FALSE	FALSE	0.053	0.689	0.652	-0.83	5.624	0.178	0.215	0.190	0.061	1.547	0.38	0.43	0.4	0.619	4	3
Locus 3	5p13.3	rs7724792	chr5	32766466	A	G	4.91	0.01	0.13	5	FALSE	TRUE	FALSE	FALSE	0.00	0.00	FALSE	FALSE	FALSE	0.052	-1.245	-1.766	-0.99	4.203	-0.263	-0.203	0.038	0.081	0.924	0.25	0.29	0.19	0.003	15	9
Locus 3	5p13.3	rs7730564	chr5	32767727	A	G	4.91	0.01	0.02	6	FALSE	FALSE	TRUE	FALSE	0.00	0.52	TRUE	FALSE	FALSE	0.263	0.738	1.447	-0.47	4.867	0.678	-0.022	0.639	0.061	0.949	0.4	0.31	0.31	0.184	1	1
Locus 2	5p13.3	rs817901	chr5	32740543	T	A	5.18	0.13	0.18	7	FALSE	FALSE	FALSE	FALSE	0.00	0.00	FALSE	FALSE	FALSE	0.044	-1.546	-2.923	-0.85	1.403	-0.141	-0.198	0.161	0.061	0.127	0.17	0.14	0.09	0.000	32	28
Locus 3	5p13.3	rs817902	chr5	32740736	G	T	5.04	0.01	0.00	6	FALSE	FALSE	FALSE	FALSE	0.00	1.56	TRUE	FALSE	FALSE	0.095	0.144	-1.169	-0.75	3.373	0.478	-0.085	0.614	0.061	0.750	0.18	0.1	0.04	0.009	6	5
Locus 2	5p13.3	rs817902	chr5	32740736	G	T	5.17	0.12	0.00	6	FALSE	FALSE	FALSE	FALSE	0.00	1.56	TRUE	FALSE	FALSE	0.095	0.144	-1.169	-0.75	3.373	0.478	-0.085	0.614	0.061	0.750	0.18	0.1	0.04	0.009	6	5
Locus 2	5p13.3	rs1698027	chr5	32738654	G	A	5.14	0.11	0.13	5	FALSE	TRUE	FALSE	FALSE	0.00	0.00	FALSE	FALSE	FALSE	0.068	-0.842	-1.664	-0.83	4.184	0.088	-0.157	0.197	0.061	0.750	0.17	0.25	0.21	0.003	18	10
Locus 3	5p13.3	rs817905	chr5	32729382	A	G	5.10	0.04	0.22	6	FALSE	FALSE	FALSE	FALSE	0.00	0.45	TRUE	FALSE	FALSE	0.044	-1.810	-3.043	-0.93	1.885	-0.322	-0.218	0.051	0.093	0.127	0.18	0.06	0.01	0.018	26	32
Locus 2	5p13.3	rs817905	chr5	32729382	A	G	5.27	0.21	0.22	6	FALSE	FALSE	FALSE	FALSE	0.00	0.45	TRUE	FALSE	FALSE	0.044	-1.810	-3.043	-0.93	1.885	-0.322	-0.218	0.051	0.093	0.127	0.18	0.06	0.01	0.018	26	32
Locus 2	5p13.3	rs864671	chr5	32729082	A	T	5.27	0.21	0.09	6	FALSE	FALSE	FALSE	FALSE	0.00	0.28	TRUE	FALSE	FALSE	0.043	-2.375	-4.143	-1.04	0.975	-0.906	-0.278	0.028	0.093	0.127	0.19	0.04	0.01	0.000	40	40
Locus 3	5p13.3	rs864671	chr5	32729082	A	T	5.10	0.04	0.09	6	FALSE	FALSE	FALSE	FALSE	0.00	0.28	TRUE	FALSE	FALSE	0.043	-2.375	-4.143	-1.04	0.975	-0.906	-0.278	0.028	0.093	0.127	0.19	0.04	0.01	0.000	40	40
Locus 3	5p13.3	rs973079	chr5	32749592	G	A	4.57	0.02	0.18	7	FALSE	FALSE	FALSE	FALSE	0.00	0.00	FALSE	FALSE	FALSE	0.045	-2.477	-3.627	-0.98	2.317	-1.023	-0.313	0.055	0.061	0.127	0.28	0.16	0.02	0.000	41	41

Supplementary Table 4. Results of the colocalization analysis

Reference locus	Compared GWAS	Source of the compared GWAS	Description of the compared GWAS	Shared SNPs	H0	H1	H2	H3	H4	n_Events	n_Total
Locus 1	pheno.395.2.GATE	UKB GATE	Nonrheumatic aortic valve disorders	2422	0.12	0.39	0.07	0.21	0.21	3435	389441
Locus 1	pheno.454.11.GATE	UKB GATE	Varicose veins of lower extremity, symptomtic	2422	0.19	0.60	0.03	0.10	0.08	856	365238
Locus 1	pheno.766.GATE	UKB GATE	Neuralgia, neuritis, and radiculitis NOS	2422	0.15	0.47	0.08	0.25	0.05	1563	403696
Locus 1	pheno.427.8.GATE	UKB GATE	Sinoatrial node dysfunction (Bradycardia)	2422	0.19	0.61	0.03	0.11	0.05	575	373492
Locus 1	pheno.791.GATE	UKB GATE	Gangrene	2422	0.20	0.65	0.02	0.08	0.05	776	407806
Locus 1	pheno.426.24.GATE	UKB GATE	Atrioventricular block, complete	2422	0.19	0.60	0.04	0.13	0.04	825	373492
Locus 1	pheno.427.6.GATE	UKB GATE	Premature beats	2422	0.19	0.62	0.04	0.12	0.04	746	373492
Locus 1	pheno.793.2.GATE	UKB GATE	Nonspecific abnormal findings on radiological and other examination of other intrathoracic organs (echocardiogram, etc)	2422	0.20	0.63	0.03	0.10	0.04	747	407656
Locus 1	pheno.765.GATE	UKB GATE	Cervical radiculitis	2422	0.20	0.64	0.03	0.09	0.04	622	403696
Locus 1	pheno.446.5.GATE	UKB GATE	Giant cell arteritis	2422	0.20	0.64	0.03	0.09	0.04	517	396788
Locus 1	pheno.454.GATE	UKB GATE	Varicose veins	2422	0.00	0.00	0.23	0.74	0.03	13156	365238
Locus 1	pheno.454.1.GATE	UKB GATE	Varicose veins of lower extremity	2422	0.00	0.00	0.23	0.74	0.03	12611	365238
Locus 1	pheno.798.1.GATE	UKB GATE	Chronic fatigue syndrome	2422	0.14	0.45	0.09	0.29	0.03	723	404385
Locus 1	pheno.426.32.GATE	UKB GATE	Left bundle branch block	2422	0.15	0.47	0.08	0.27	0.03	2482	373492
Locus 1	pheno.425.1.GATE	UKB GATE	Primary/intrinsic cardiomyopathies	2422	0.19	0.60	0.04	0.14	0.03	1620	404327
Locus 1	pheno.430.2.GATE	UKB GATE	Intracerebral hemorrhage	2422	0.19	0.62	0.04	0.12	0.03	1018	395927
Locus 1	pheno.415.21.GATE	UKB GATE	Primary pulmonary hypertension	2422	0.19	0.62	0.04	0.12	0.03	502	399568
Locus 1	pheno.447.1.GATE	UKB GATE	Stricture of artery	2422	0.20	0.64	0.03	0.11	0.03	1038	396788
Locus 1	pheno.446.GATE	UKB GATE	Polyarteritis nodosa and allied conditions	2422	0.20	0.63	0.03	0.11	0.03	1018	396788
Locus 1	pheno.426.23.GATE	UKB GATE	Second degree AV block	2422	0.20	0.64	0.03	0.10	0.03	623	373492
Locus 1	pheno.771.GATE	UKB GATE	Musculoskeletal symptoms referable to limbs	2422	0.20	0.64	0.03	0.09	0.03	960	401991
Locus 1	pheno.772.GATE	UKB GATE	Symptoms of the muscles	2422	0.20	0.65	0.03	0.09	0.03	838	407617
Locus 1	pheno.416.GATE	UKB GATE	Cardiomegaly	2422	0.21	0.66	0.03	0.08	0.03	3758	399568
Locus 1	pheno.444.GATE	UKB GATE	Arterial embolism and thrombosis	2422	0.21	0.66	0.03	0.08	0.03	1099	396788
Locus 1	pheno.452.GATE	UKB GATE	Other venous embolism and thrombosis	2422	0.21	0.66	0.02	0.08	0.03	724	365238
Locus 1	pheno.444.1.GATE	UKB GATE	Arterial embolism and thrombosis of lower extremity artery	2422	0.21	0.66	0.03	0.08	0.03	662	396788
Locus 1	pheno.790.6.GATE	UKB GATE	Other abnormal blood chemistry	2422	0.21	0.67	0.02	0.07	0.03	8303	400012
Locus 1	pheno.426.91.GATE	UKB GATE	Cardiac pacemaker in situ	2422	0.21	0.67	0.02	0.07	0.03	3082	373492
Locus 1	pheno.433.1.GATE	UKB GATE	Occlusion and stenosis of precerebral arteries	2422	0.21	0.67	0.02	0.07	0.03	1658	395927
Locus 1	pheno.440.2.GATE	UKB GATE	Atherosclerosis of the extremities	2422	0.21	0.68	0.02	0.07	0.03	1032	396788
Locus 1	pheno.450.GATE	UKB GATE	Noninfectious disorders of lymphatic channels	2422	0.21	0.67	0.02	0.07	0.03	978	407604
Locus 1	pheno.401.2.GATE	UKB GATE	Hypertensive heart and/or renal disease	2422	0.21	0.68	0.02	0.06	0.03	2048	315595
Locus 1	pheno.433.8.GATE	UKB GATE	Late effects of cerebrovascular disease	2422	0.21	0.67	0.02	0.06	0.03	1640	395927
Locus 1	pheno.785.GATE	UKB GATE	Abdominal pain	2422	0.22	0.71	0.01	0.02	0.03	46029	362553
Locus 1	pheno.451.GATE	UKB GATE	Phlebitis and thrombophlebitis	2422	0.08	0.26	0.15	0.48	0.02	4779	365238
Locus 1	pheno.451.2.GATE	UKB GATE	Phlebitis and thrombophlebitis of lower extremities	2422	0.09	0.29	0.14	0.45	0.02	4406	365238
Locus 1	pheno.782.3.GATE	UKB GATE	Edema	2422	0.17	0.53	0.07	0.22	0.02	2408	405827
Locus 1	pheno.395.1.GATE	UKB GATE	Nonrheumatic mitral valve disorders	2422	0.18	0.56	0.06	0.19	0.02	3088	389441
Locus 1	pheno.411.9.GATE	UKB GATE	Other acute and subacute forms of ischemic heart disease	2422	0.17	0.55	0.06	0.19	0.02	1983	370814
Locus 1	pheno.458.1.GATE	UKB GATE	Orthostatic hypotension	2422	0.18	0.56	0.06	0.18	0.02	1981	381440
Locus 1	pheno.764.GATE	UKB GATE	Sciatica	2422	0.18	0.58	0.05	0.17	0.02	2906	403696
Locus 1	pheno.426.21.GATE	UKB GATE	First degree AV block	2422	0.19	0.60	0.05	0.15	0.02	1481	373492
Locus 1	pheno.415.2.GATE	UKB GATE	Chronic pulmonary heart disease	2422	0.20	0.62	0.04	0.12	0.02	724	399568
Locus 1	pheno.420.3.GATE	UKB GATE	Endocarditis	2422	0.20	0.63	0.04	0.11	0.02	917	404327
Locus 1	pheno.426.3.GATE	UKB GATE	Bundle branch block	2422	0.20	0.65	0.03	0.10	0.02	4663	373492
Locus 1	pheno.798.GATE	UKB GATE	Malaise and fatigue	2422	0.20	0.65	0.03	0.10	0.02	4197	404385
Locus 1	pheno.401.22.GATE	UKB GATE	Hypertensive chronic kidney disease	2422	0.20	0.65	0.03	0.09	0.02	1583	315595
Locus 1	pheno.441.GATE	UKB GATE	Vascular insufficiency of intestine	2422	0.21	0.66	0.03	0.09	0.02	1429	396788
Locus 1	pheno.426.2.GATE	UKB GATE	Atrioventricular [AV] block	2422	0.21	0.67	0.03	0.08	0.02	3012	373492
Locus 1	pheno.433.31.GATE	UKB GATE	Transient cerebral ischemia	2422	0.21	0.67	0.02	0.08	0.02	2559	395927
Locus 1	pheno.429.GATE	UKB GATE	Ill-defined descriptions and complications of heart disease	2422	0.21	0.66	0.03	0.08	0.02	1028	399998
Locus 1	pheno.430.GATE	UKB GATE	Intracranial hemorrhage	2422	0.21	0.68	0.02	0.07	0.02	2393	395927
Locus 1	pheno.761.GATE	UKB GATE	Cervicalgia	2422	0.21	0.68	0.02	0.07	0.02	2157	406425
Locus 1	pheno.395.6.GATE	UKB GATE	Heart valve replaced	2422	0.21	0.68	0.02	0.07	0.02	1891	389441
Locus 1	pheno.402.GATE	UKB GATE	Elevated blood pressure reading without diagnosis of hypertension	2422	0.21	0.68	0.02	0.07	0.02	1793	315595
Locus 1	pheno.770.GATE	UKB GATE	Myalgia and myositis unspecified	2422	0.21	0.67	0.02	0.07	0.02	909	407673
Locus 1	pheno.788.GATE	UKB GATE	Syncope and collapse	2422	0.21	0.68	0.02	0.06	0.02	11041	397541
Locus 1	pheno.426.9.GATE	UKB GATE	Cardiac pacemaker/device in situ	2422	0.22	0.69	0.02	0.06	0.02	3332	373492
Locus 1	pheno.443.1.GATE	UKB GATE	Raynaud's syndrome	2422	0.21	0.68	0.02	0.06	0.02	1471	396788
Locus 1	pheno.442.GATE	UKB GATE	Other aneurysm	2422	0.22	0.69	0.02	0.05	0.02	2545	396788
Locus 1	pheno.433.21.GATE	UKB GATE	Cerebral artery occlusion, with cerebral infarction	2422	0.22	0.69	0.02	0.05	0.02	2063	395927
Locus 1	pheno.440.GATE	UKB GATE	Atherosclerosis	2422	0.22	0.70	0.02	0.05	0.02	1781	396788
Locus 1	pheno.783.GATE	UKB GATE	Fever of unknown origin	2422	0.22	0.71	0.01	0.04	0.02	4869	403713
Locus 1	pheno.411.8.GATE	UKB GATE	Other chronic ischemic heart disease, unspecified	2422	0.17	0.54	0.07	0.21	0.01	30853	370814
Locus 1	pheno.415.1.GATE	UKB GATE	Acute pulmonary heart disease	2422	0.18	0.56	0.06	0.19	0.01	4940	399568
Locus 1	pheno.415.11.GATE	UKB GATE	Pulmonary embolism and infarction, acute	2422	0.18	0.56	0.06	0.19	0.01	4940	399568
Locus 1	pheno.773.GATE	UKB GATE	Pain in limb	2422	0.19	0.62	0.04	0.13	0.01	7405	401177
Locus 1	pheno.428.2.GATE	UKB GATE	Heart failure NOS	2422	0.21	0.66	0.03	0.10	0.01	7624	399998
Locus 1	pheno.411.1.GATE	UKB GATE	Unstable angina (intermediate coronary syndrome)	2422	0.21	0.67	0.03	0.08	0.01	5505	370814
Locus 1	pheno.411.3.GATE	UKB GATE	Angina pectoris	2422	0.21	0.68	0.02	0.07	0.01	20875	370814
Locus 1	pheno.415.GATE	UKB GATE	Pulmonary heart disease	2422	0.22	0.69	0.02	0.07	0.01	5635	399568
Locus 1	pheno.443.9.GATE	UKB GATE	Peripheral vascular disease, unspecified	2422	0.21	0.68	0.02	0.07	0.01	3298	396788
Locus 1	pheno.411.2.GATE	UKB GATE	Myocardial infarction	2422	0.22	0.70	0.02	0.06	0.01	14781	370814
Locus 1	pheno.433.2.GATE	UKB GATE	Occlusion of cerebral arteries	2422	0.22	0.69	0.02	0.06	0.01	5339	395927
Locus 1	pheno.418.1.GATE	UKB GATE	Precordial pain	2422	0.22	0.71	0.01	0.05	0.01	4507	373568
Locus 1	pheno.427.GATE	UKB GATE	Cardiac dysrhythmias	2422	0.23	0.72	0.01	0.04	0.01	30709	373492
Locus 1	pheno.459.9.GATE	UKB GATE	Circulatory disease NEC	2422	0.22	0.72	0.01	0.04	0.01	20772	381440
Locus 1	pheno.771.1.GATE	UKB GATE	Swelling of limb	2422	0.22	0.71	0.01	0.04	0.01	5688	401991
Locus 1	pheno.433.3.GATE	UKB GATE	Cerebral ischemia	2422	0.22	0.72	0.01	0.04	0.01	5602	395927
Locus 1	pheno.443.GATE	UKB GATE	Peripheral vascular disease	2422	0.22	0.71	0.01	0.04	0.01	4970	396788
Locus 1	pheno.427.9.GATE	UKB GATE	Palpitations	2422	0.22	0.71	0.01	0.04	0.01	4514	373492
Locus 1	pheno.455.GATE	UKB GATE	Hemorrhoids	2422	0.23	0.73	0.01	0.03	0.01	26034	365238
Locus 1	pheno.459.GATE	UKB GATE	Other disorders of circulatory system	2422	0.23	0.72	0.01	0.03	0.01	21015	381440
Locus 1	pheno.396.GATE	UKB GATE	Abnormal heart sounds	2422	0.23	0.73	0.01	0.03	0.01	11861	389441
Locus 1	pheno.433.GATE	UKB GATE	Cerebrovascular disease	2422	0.23	0.72	0.01	0.03	0.01	11116	395927
Locus 1	pheno.781.GATE	UKB GATE	Symptoms involving nervous and musculoskeletal systems	2422	0.23	0.72	0.01	0.03	0.01	8903	399677
Locus 1	pheno.458.GATE	UKB GATE	Hypotension	2422	0.23	0.72	0.01	0.03	0.01	8152	381440
Locus 1	pheno.458.9.GATE	UKB GATE	Hypotension NOS	2422	0.23	0.72	0.01	0.03	0.01	6949	381440
Locus 1	pheno.418.GATE	UKB GATE	Nonspecific chest pain	2422	0.23	0.74	0.01	0.02	0.01	32343	373568
Locus 1	pheno.401.GATE	UKB GATE	Hypertension	2422	0.23	0.74	0.01	0.02	0.00	92267	315595
Locus 1	pheno.401.1.GATE	UKB GATE	Essential hypertension	2422	0.23	0.74	0.01	0.02	0.00	91962	315595
Locus 1	COVID19_HGI_A2_ALL_leave_UKBB_23andme_20210107	HGI	Very severe respiratory confirmed covid vs. population	2316	0.22	0.70	0.01	0.04	0.03	5870	1155203
Locus 1	COVID19_HGI_B1_ALL_eur_leave_23andme_20210107	HGI	Hospitalized covid vs. not hospitalized covid (EUR)	2205	0.22	0.68	0.02	0.06	0.02	4829	11816
Locus 1	COVID19_HGI_A2_ALL_leave_23andme_20210107	HGI	Very severe respiratory confirmed covid vs. population	2306	0.22	0.70	0.01	0.05	0.02	5582	709010
Locus 1	COVID19_HGI_B1_ALL_leave_23andme_20210107	HGI	Hospitalized covid vs. not hospitalized covid	2242	0.23	0.69	0.01	0.04	0.02	5773	15497
Locus 1	COVID19_HGI_A2_ALL_eur_leave_ukbb_23andme_20210107	HGI	Very severe respiratory confirmed covid vs. population (EUR)	2432	0.22	0.71	0.01	0.04	0.02	4792	1054664
Locus 1	COVID19_HGI_A2_ALL_eur_leave_23andme_20210107	HGI	Very severe respiratory confirmed covid vs. population (EUR)	2420	0.22	0.71	0.01	0.05	0.01	5101	1383241
Locus 1	COVID19_HGI_B1_ALL_eur_leave_ukbb_23andme_20210107	HGI	Hospitalized covid vs. not hospitalized covid (EUR)	2206	0.23	0.69	0.02	0.05	0.01	3159	7206
Locus 1	COVID19_HGI_B1_ALL_leave_UKBB_23andme_20210107	HGI	Hospitalized covid vs. not hospitalized covid	2105	0.23	0.70	0.01	0.04	0.01	3961	10538
Locus 1	COVID19_HGI_B2_ALL_leave_23andme_20210107	HGI	Hospitalized covid vs. population	2307	0.23	0.73	0.01	0.03	0.01	12888	1295966
Locus 1	COVID19_HGI_B2_ALL_eur_leave_23andme_20210107	HGI	Hospitalized covid vs. population (EUR)	2275	0.23	0.72	0.01	0.03	0.01	9986	1877672
Locus 1	COVID19_HGI_B2_ALL_eur_leave_ukbb_23andme_20210107	HGI	Hospitalized covid vs. population (EUR)	2277	0.23	0.71	0.01	0.03	0.01	8316	1549095
Locus 1	COVID19_HGI_C2_ALL_eur_leave_ukbb_23andme_20210107	HGI	Covid vs. population (EUR)	2396	0.23	0.74	0.01	0.02	0.01	32494	1316207
Locus 1	COVID19_HGI_B2_ALL_leave_UKBB_23andme_20210107	HGI	Hospitalized covid vs. population	2167	0.24	0.72	0.01	0.02	0.01	11829	1725210
Locus 1	COVID19_HGI_C2_ALL_eur_leave_23andme_20210107	HGI	Covid vs. population (EUR)	2395	0.23	0.72	0.01	0.04	0.00	38984	1644784
Locus 1	COVID19_HGI_C2_ALL_leave_23andme_20210107	HGI	Covid vs. population	2369	0.23	0.72	0.01	0.04	0.00	36590	1668938
Locus 1	COVID19_HGI_C2_ALL_leave_UKBB_23andme_20210107	HGI	Covid vs. population	2335	0.23	0.74	0.01	0.02	0.00	42557	1424707
Locus 1	ENSG00000157693.10 in Artery_Tibial	GTEx cis-eQTL	ENSG00000157693.10 in Artery_Tibial	1453	0.30	0.42	0.06	0.08	0.14	NA	388
Locus 1	ENSG00000041982.10 in Artery_Tibial	GTEx cis-eQTL	ENSG00000041982.10 in Artery_Tibial	2319	0.16	0.52	0.05	0.16	0.11	NA	388
Locus 1	ENSG00000181634.7 in Artery_Aorta	GTEx cis-eQTL	ENSG00000181634.7 in Artery_Aorta	1840	0.19	0.59	0.03	0.08	0.11	NA	267
Locus 1	ENSG00000041982.10 in Artery_Aorta	GTEx cis-eQTL	ENSG00000041982.10 in Artery_Aorta	2319	0.17	0.55	0.04	0.13	0.10	NA	267
Locus 1	ENSG00000136888.6 in Artery_Tibial	GTEx cis-eQTL	ENSG00000136888.6 in Artery_Tibial	1397	0.34	0.47	0.04	0.05	0.10	NA	388
Locus 1	ENSG00000173077.10 in Artery_Coronary	GTEx cis-eQTL	ENSG00000173077.10 in Artery_Coronary	2338	0.18	0.57	0.04	0.13	0.08	NA	152
Locus 1	ENSG00000136888.6 in Artery_Coronary	GTEx cis-eQTL	ENSG00000136888.6 in Artery_Coronary	1397	0.35	0.47	0.04	0.06	0.08	NA	152
Locus 1	ENSG00000234692.1 in Artery_Tibial	GTEx cis-eQTL	ENSG00000234692.1 in Artery_Tibial	2342	0.00	0.00	0.22	0.71	0.07	NA	388
Locus 1	ENSG00000106952.3 in Artery_Coronary	GTEx cis-eQTL	ENSG00000106952.3 in Artery_Coronary	2133	0.16	0.52	0.06	0.19	0.07	NA	152
Locus 1	ENSG00000106952.3 in Whole_Blood	GTEx cis-eQTL	ENSG00000106952.3 in Whole_Blood	2133	0.19	0.59	0.04	0.12	0.07	NA	369
Locus 1	ENSG00000182752.8 in Artery_Coronary	GTEx cis-eQTL	ENSG00000182752.8 in Artery_Coronary	2326	0.18	0.59	0.04	0.13	0.06	NA	152
Locus 1	ENSG00000182752.8 in Artery_Aorta	GTEx cis-eQTL	ENSG00000182752.8 in Artery_Aorta	2326	0.18	0.59	0.04	0.13	0.06	NA	267
Locus 1	ENSG00000041982.10 in Artery_Coronary	GTEx cis-eQTL	ENSG00000041982.10 in Artery_Coronary	2319	0.19	0.59	0.04	0.13	0.05	NA	152
Locus 1	ENSG00000041982.10 in Heart_Left_Ventricle	GTEx cis-eQTL	ENSG00000041982.10 in Heart_Left_Ventricle	2319	0.19	0.61	0.04	0.12	0.05	NA	272

Locus 1	ENSG00000181634.7 in Artery_Tibial	GTEx cis-eQTL	ENSG00000181634.7 in Artery_Tibial	1840	0.20	0.62	0.03	0.10	0.05	NA	388
Locus 1	ENSG00000106952.3 in Heart_Left_Ventricle	GTEx cis-eQTL	ENSG00000106952.3 in Heart_Left_Ventricle	2133	0.20	0.62	0.03	0.10	0.05	NA	272
Locus 1	ENSG00000181634.7 in Artery_Coronary	GTEx cis-eQTL	ENSG00000181634.7 in Artery_Coronary	1840	0.20	0.62	0.03	0.10	0.05	NA	152
Locus 1	ENSG00000106952.3 in Artery_Tibial	GTEx cis-eQTL	ENSG00000106952.3 in Artery_Tibial	2133	0.20	0.62	0.03	0.10	0.05	NA	388
Locus 1	ENSG00000157693.10 in Artery_Coronary	GTEx cis-eQTL	ENSG00000157693.10 in Artery_Coronary	1453	0.35	0.49	0.05	0.07	0.05	NA	152
Locus 1	ENSG00000173077.10 in Artery_Tibial	GTEx cis-eQTL	ENSG00000173077.10 in Artery_Tibial	2338	0.17	0.54	0.06	0.20	0.04	NA	388
Locus 1	ENSG00000041982.10 in Whole_Blood	GTEx cis-eQTL	ENSG00000041982.10 in Whole_Blood	2319	0.17	0.55	0.06	0.19	0.04	NA	369
Locus 1	ENSG00000182752.8 in Heart_Left_Ventricle	GTEx cis-eQTL	ENSG00000182752.8 in Heart_Left_Ventricle	2326	0.19	0.60	0.04	0.13	0.04	NA	272
Locus 1	ENSG00000106952.3 in Artery_Aorta	GTEx cis-eQTL	ENSG00000106952.3 in Artery_Aorta	2133	0.19	0.60	0.04	0.12	0.04	NA	267
Locus 1	ENSG00000182752.8 in Artery_Tibial	GTEx cis-eQTL	ENSG00000182752.8 in Artery_Tibial	2326	0.19	0.62	0.04	0.11	0.04	NA	388
Locus 1	ENSG00000181634.7 in Whole_Blood	GTEx cis-eQTL	ENSG00000181634.7 in Whole_Blood	1840	0.20	0.64	0.03	0.08	0.04	NA	369
Locus 1	ENSG00000136888.6 in Heart_Left_Ventricle	GTEx cis-eQTL	ENSG00000136888.6 in Heart_Left_Ventricle	1397	0.36	0.49	0.05	0.07	0.04	NA	272
Locus 1	ENSG00000157693.10 in Artery_Aorta	GTEx cis-eQTL	ENSG00000157693.10 in Artery_Aorta	1453	0.35	0.50	0.05	0.07	0.04	NA	267
Locus 1	ENSG00000157693.10 in Whole_Blood	GTEx cis-eQTL	ENSG00000157693.10 in Whole_Blood	1453	0.35	0.49	0.05	0.07	0.04	NA	369
Locus 1	ENSG00000136888.6 in Whole_Blood	GTEx cis-eQTL	ENSG00000136888.6 in Whole_Blood	1397	0.37	0.51	0.03	0.05	0.04	NA	369
Locus 1	ENSG00000157693.10 in Heart_Left_Ventricle	GTEx cis-eQTL	ENSG00000157693.10 in Heart_Left_Ventricle	1453	0.34	0.48	0.06	0.09	0.03	NA	272
Locus 1	ENSG00000136888.6 in Artery_Aorta	GTEx cis-eQTL	ENSG00000136888.6 in Artery_Aorta	1397	0.37	0.50	0.04	0.05	0.03	NA	267
Locus 1	ENSG00000095397.9 in Whole_Blood	GTEx cis-eQTL	ENSG00000095397.9 in Whole_Blood	1053	0.74	0.15	0.07	0.01	0.02	NA	369
Locus 1	ENSG00000095397.9 in Artery_Tibial	GTEx cis-eQTL	ENSG00000095397.9 in Artery_Tibial	1053	0.73	0.15	0.10	0.02	0.01	NA	388
Locus 1	ENSG00000095397.9 in Artery_Aorta	GTEx cis-eQTL	ENSG00000095397.9 in Artery_Aorta	1053	0.74	0.15	0.08	0.02	0.01	NA	267
Locus 1	ENSG00000095397.9 in Artery_Coronary	GTEx cis-eQTL	ENSG00000095397.9 in Artery_Coronary	1053	0.76	0.15	0.07	0.01	0.01	NA	152
Locus 1	ENSG00000095397.9 in Heart_Left_Ventricle	GTEx cis-eQTL	ENSG00000095397.9 in Heart_Left_Ventricle	1053	0.76	0.15	0.06	0.01	0.01	NA	272
Locus 1	ENSG00000235119.1 in Artery_Aorta	GTEx cis-eQTL	ENSG00000235119.1 in Artery_Aorta	711	0.79	0.06	0.13	0.01	0.01	NA	267
Locus 1	ENSG00000119401.10 in Artery_Aorta	GTEx cis-eQTL	ENSG00000119401.10 in Artery_Aorta	736	0.84	0.08	0.07	0.01	0.01	NA	267
Locus 1	ENSG00000235119.1 in Artery_Coronary	GTEx cis-eQTL	ENSG00000235119.1 in Artery_Coronary	711	0.84	0.07	0.08	0.01	0.01	NA	152
Locus 1	ENSG00000119401.10 in Whole_Blood	GTEx cis-eQTL	ENSG00000119401.10 in Whole_Blood	736	0.85	0.08	0.06	0.01	0.01	NA	369
Locus 1	ENSG00000235119.1 in Artery_Tibial	GTEx cis-eQTL	ENSG00000235119.1 in Artery_Tibial	711	0.85	0.07	0.07	0.01	0.01	NA	388
Locus 1	ENSG00000119401.10 in Artery_Tibial	GTEx cis-eQTL	ENSG00000119401.10 in Artery_Tibial	736	0.85	0.08	0.06	0.01	0.01	NA	388
Locus 1	ENSG00000119401.10 in Artery_Coronary	GTEx cis-eQTL	ENSG00000119401.10 in Artery_Coronary	736	0.85	0.08	0.05	0.00	0.01	NA	152
Locus 1	ENSG00000119401.10 in Heart_Left_Ventricle	GTEx cis-eQTL	ENSG00000119401.10 in Heart_Left_Ventricle	736	0.86	0.08	0.05	0.00	0.01	NA	272
Locus 1	ENSG00000106948.12 in Artery_Coronary	GTEx cis-eQTL	ENSG00000106948.12 in Artery_Coronary	705	0.87	0.07	0.06	0.00	0.01	NA	152
Locus 1	ENSG00000106948.12 in Artery_Tibial	GTEx cis-eQTL	ENSG00000106948.12 in Artery_Tibial	705	0.87	0.07	0.05	0.00	0.01	NA	388
Locus 1	ENSG00000235119.1 in Whole_Blood	GTEx cis-eQTL	ENSG00000235119.1 in Whole_Blood	711	0.87	0.07	0.05	0.00	0.01	NA	369
Locus 1	ENSG00000106948.12 in Whole_Blood	GTEx cis-eQTL	ENSG00000106948.12 in Whole_Blood	705	0.87	0.07	0.05	0.00	0.01	NA	369
Locus 1	ENSG00000106948.12 in Artery_Aorta	GTEx cis-eQTL	ENSG00000106948.12 in Artery_Aorta	705	0.87	0.07	0.05	0.00	0.01	NA	267
Locus 1	ENSG00000229314.4 in Artery_Tibial	GTEx cis-eQTL	ENSG00000229314.4 in Artery_Tibial	447	0.88	0.04	0.07	0.00	0.01	NA	388
Locus 1	ENSG00000229314.4 in Whole_Blood	GTEx cis-eQTL	ENSG00000229314.4 in Whole_Blood	447	0.91	0.04	0.04	0.00	0.01	NA	369
Locus 1	ENSG00000229314.4 in Heart_Left_Ventricle	GTEx cis-eQTL	ENSG00000229314.4 in Heart_Left_Ventricle	447	0.91	0.04	0.04	0.00	0.01	NA	272
Locus 1	ENSG00000228278.2 in Heart_Left_Ventricle	GTEx cis-eQTL	ENSG00000228278.2 in Heart_Left_Ventricle	465	0.91	0.04	0.04	0.00	0.01	NA	272
Locus 1	ENSG00000228278.2 in Whole_Blood	GTEx cis-eQTL	ENSG00000228278.2 in Whole_Blood	465	0.91	0.04	0.04	0.00	0.01	NA	369
Locus 1	ENSG00000106948.12 in Heart_Left_Ventricle	GTEx cis-eQTL	ENSG00000106948.12 in Heart_Left_Ventricle	705	0.88	0.07	0.04	0.00	0.00	NA	272
Locus 1	ENSG00000228278.2 in Artery_Tibial	GTEx cis-eQTL	ENSG00000228278.2 in Artery_Tibial	465	0.90	0.04	0.05	0.00	0.00	NA	388
Locus 1	ENSG00000228278.2 in Artery_Coronary	GTEx cis-eQTL	ENSG00000228278.2 in Artery_Coronary	465	0.91	0.04	0.04	0.00	0.00	NA	152
Locus 1	ENSG00000229314.4 in Artery_Coronary	GTEx cis-eQTL	ENSG00000229314.4 in Artery_Coronary	447	0.91	0.04	0.04	0.00	0.00	NA	152
Locus 1	ENSG00000229314.4 in Artery_Aorta	GTEx cis-eQTL	ENSG00000229314.4 in Artery_Aorta	447	0.92	0.04	0.04	0.00	0.00	NA	267
Locus 1	ENSG00000228278.2 in Artery_Aorta	GTEx cis-eQTL	ENSG00000228278.2 in Artery_Aorta	465	0.92	0.04	0.03	0.00	0.00	NA	267
Locus 1	ENSG00000196739.10 in Artery_Coronary	GTEx cis-eQTL	ENSG00000196739.10 in Artery_Coronary	19	1.00	0.00	0.00	0.00	0.00	NA	152
Locus 1	ENSG00000196739.10 in Heart_Left_Ventricle	GTEx cis-eQTL	ENSG00000196739.10 in Heart_Left_Ventricle	19	1.00	0.00	0.00	0.00	0.00	NA	272
Locus 1	ENSG00000196739.10 in Artery_Aorta	GTEx cis-eQTL	ENSG00000196739.10 in Artery_Aorta	19	1.00	0.00	0.00	0.00	0.00	NA	267
Locus 1	ENSG00000196739.10 in Artery_Tibial	GTEx cis-eQTL	ENSG00000196739.10 in Artery_Tibial	19	1.00	0.00	0.00	0.00	0.00	NA	388
Locus 1	ENSG00000196739.10 in Whole_Blood	GTEx cis-eQTL	ENSG00000196739.10 in Whole_Blood	19	1.00	0.00	0.00	0.00	0.00	NA	369
Locus 1	finngen_R4_I9_CARDMYOHYP	FinnGen	Cardiomyopathy, Hypertrophic obstructive	2324	0.10	0.31	0.07	0.21	0.31	251	130159
Locus 1	finngen_R4_I9_CARDMYO	FinnGen	Cardiomyopathy	2324	0.19	0.58	0.02	0.08	0.13	2342	132250
Locus 1	finngen_R4_I9_CARDARR	FinnGen	Cardiac arrest	2324	0.18	0.57	0.04	0.14	0.07	772	97986
Locus 1	finngen_R4_FG_CARDMYO	FinnGen	Cardiomyopathy (excluding other)	2324	0.21	0.65	0.02	0.08	0.05	1730	134693
Locus 1	finngen_R4_I9_CARDMPRI	FinnGen	Cardiomyopathies, Primary/intrinsic	2324	0.20	0.64	0.03	0.08	0.05	1678	131586
Locus 1	finngen_R4_I9_CARDMYOOTH	FinnGen	Cardiomyopathy, other and unspecified	2324	0.20	0.63	0.03	0.08	0.05	971	130879
Locus 1	finngen_R4_I9_OTHILLHEART	FinnGen	Other or ill-defined heart diseases	2324	0.20	0.64	0.03	0.09	0.04	583	130491
Locus 1	finngen_R4_I9_K_CARDIAC	FinnGen	Death due to cardiac causes	2324	0.22	0.68	0.02	0.05	0.04	6314	176899
Locus 1	finngen_R4_I9_MYOCARD	FinnGen	Myocarditis	2324	0.19	0.59	0.04	0.14	0.03	626	97840
Locus 1	finngen_R4_FG_OTH	FinnGen	Other CVD (FINNGEN)	2324	0.20	0.64	0.03	0.10	0.03	1398	98612
Locus 1	finngen_R4_I9_NONISCHCARDMYOP_STRICT	FinnGen	Nonischemic cardiomyopathy	2324	0.21	0.66	0.02	0.07	0.03	653	151314
Locus 1	finngen_R4_I9_OTH	FinnGen	Other CVD	2324	0.20	0.62	0.04	0.12	0.02	1721	176899
Locus 1	finngen_R4_I9_CABG	FinnGen	Coronary artery bypass grafting	2324	0.21	0.67	0.02	0.08	0.02	4449	171377
Locus 1	finngen_R4_I9_HYPTENSHR	FinnGen	Hypertensive heart and/or renal disease	2324	0.22	0.68	0.02	0.07	0.02	3674	136997
Locus 1	finngen_R4_I9_HYPTENSHD	FinnGen	Hypertensive Heart Disease	2324	0.22	0.69	0.02	0.05	0.02	3252	136575
Locus 1	finngen_R4_I9_HEARTFAIL	FinnGen	Heart failure,strict	2324	0.23	0.71	0.01	0.04	0.02	9576	168862
Locus 1	finngen_R4_I9_CVD	FinnGen	Cardiovascular diseases	2324	0.23	0.71	0.01	0.02	0.02	86957	176899
Locus 1	finngen_R4_I9_ISCHHEART	FinnGen	Ischemic heart diseases	2324	0.23	0.72	0.01	0.02	0.02	24278	176899
Locus 1	finngen_R4_I9_ANGIO	FinnGen	Coronary angiopasty	2324	0.21	0.67	0.02	0.08	0.01	6699	173627
Locus 1	finngen_R4_FG_OTHHEART	FinnGen	Other heart diseases	2324	0.22	0.70	0.02	0.05	0.01	43936	141150
Locus 1	finngen_R4_I9_AF	FinnGen	Atrial fibrillation and flutter	2324	0.22	0.70	0.02	0.05	0.01	17325	114539
Locus 1	finngen_R4_I9_ANGINA	FinnGen	Angina pectoris	2324	0.23	0.72	0.01	0.04	0.01	14712	167333
Locus 1	finngen_R4_I9_MI	FinnGen	Myocardial infarction	2324	0.23	0.71	0.01	0.04	0.01	10003	162624
Locus 1	finngen_R4_I9_NONISCHCARDMYOP	FinnGen	Non-ischemic cardiomyopathy	2324	0.23	0.71	0.01	0.04	0.01	8300	151533
Locus 1	finngen_R4_I9_AF_REIMB	FinnGen	Atrial fibrillation and flutter with reimbursement	2324	0.23	0.71	0.01	0.04	0.01	8171	105385
Locus 1	finngen_R4_FG_CVD	FinnGen	Cardiovascular diseases (excluding rheumatic etc)	2324	0.23	0.72	0.01	0.03	0.01	79685	176899
Locus 1	finngen_R4_I9_OTHHEART	FinnGen	Other heart diseases	2324	0.23	0.72	0.01	0.03	0.01	46991	176899
Locus 1	finngen_R4_I9_CORATHER	FinnGen	Coronary atherosclerosis	2324	0.23	0.72	0.01	0.03	0.01	18295	170916
Locus 1	finngen_R4_I9_HEARTFAIL_NS	FinnGen	Heart failure, not strict	2324	0.23	0.71	0.01	0.03	0.01	17613	176899
Locus 1	finngen_R4_I9_HEARTFAIL_ALLCAUSE	FinnGen	All-cause Heart Failure	2324	0.23	0.72	0.01	0.03	0.01	17387	176445
Locus 1	finngen_R4_I9_CHD_NOREV	FinnGen	Major coronary heart disease event excluding revascularizations	2324	0.23	0.72	0.01	0.03	0.01	13809	173972
Locus 1	finngen_R4_I9_MI_STRICT	FinnGen	Myocardial infarction, strict	2324	0.23	0.72	0.01	0.03	0.01	9145	161766
Locus 1	finngen_R4_I9_HYPERTENSION	FinnGen	Hypertensive diseases	2324	0.23	0.73	0.01	0.02	0.01	43576	176899
Locus 1	finngen_R4_I9_HYPTENSESS	FinnGen	Hypertension, essential	2324	0.23	0.73	0.01	0.02	0.01	33229	166552
Locus 1	finngen_R4_I9_CVD_HARD	FinnGen	Hard cardiovascular diseases	2324	0.23	0.73	0.01	0.02	0.01	22860	176899
Locus 1	finngen_R4_I9_CHD	FinnGen	Major coronary heart disease event	2324	0.23	0.73	0.01	0.02	0.01	16631	176899
Locus 3	pheno.433.1.GATE	UKB GATE	Occlusion and stenosis of precerebral arteries	2435	0.01	0.57	0.00	0.08	0.35	1658	395927
Locus 3	pheno.443.1.GATE	UKB GATE	Raynaud's syndrome	2435	0.01	0.66	0.00	0.08	0.25	1471	396788
Locus 3	pheno.772.GATE	UKB GATE	Symptoms of the muscles	2435	0.01	0.72	0.00	0.11	0.16	838	407617
Locus 3	pheno.443.GATE	UKB GATE	Peripheral vascular disease	2435	0.01	0.79	0.00	0.07	0.13	4970	396788
Locus 3	pheno.426.23.GATE	UKB GATE	Second degree AV block	2435	0.01	0.79	0.00	0.11	0.09	623	373492
Locus 3	pheno.782.3.GATE	UKB GATE	Edema	2435	0.01	0.84	0.00	0.08	0.07	2408	405827
Locus 3	pheno.433.2.GATE	UKB GATE	Occlusion of cerebral arteries	2435	0.01	0.86	0.00	0.06	0.07	5339	395927
Locus 3	pheno.454.11.GATE	UKB GATE	Varicose veins of lower extremity, symptomtic	2435	0.01	0.66	0.00	0.27	0.06	856	365238
Locus 3	pheno.444.1.GATE	UKB GATE	Arterial embolism and thrombosis of lower extremity artery	2435	0.01	0.83	0.00	0.10	0.06	662	396788
Locus 3	pheno.798.1.GATE	UKB GATE	Chronic fatigue syndrome	2435	0.01	0.79	0.00	0.15	0.05	723	404385
Locus 3	pheno.442.GATE	UKB GATE	Other aneurysm	2435	0.01	0.80	0.00	0.14	0.05	2545	396788
Locus 3	pheno.452.GATE	UKB GATE	Other venous embolism and thrombosis	2435	0.01	0.81	0.00	0.12	0.05	724	365238
Locus 3	pheno.765.GATE	UKB GATE	Cervical radiculitis	2435	0.01	0.82	0.00	0.11	0.05	622	403696
Locus 3	pheno.427.9.GATE	UKB GATE	Palpitations	2435	0.01	0.87	0.00	0.07	0.05	4514	373492
Locus 3	pheno.427.GATE	UKB GATE	Cardiac dysrhythmias	2435	0.01	0.88	0.00	0.05	0.05	30709	373492
Locus 3	pheno.426.24.GATE	UKB GATE	Atrioventricular block, complete	2435	0.01	0.79	0.00	0.16	0.04	825	373492
Locus 3	pheno.443.9.GATE	UKB GATE	Peripheral vascular disease, unspecified	2435	0.01	0.86	0.00	0.09	0.04	3298	396788
Locus 3	pheno.440.2.GATE	UKB GATE	Atherosclerosis of the extremities	2435	0.01	0.86	0.00	0.09	0.04	1032	396788
Locus 3	pheno.430.2.GATE	UKB GATE	Intracerebral hemorrhage	2435	0.01	0.57	0.01	0.38	0.03	1018	395927
Locus 3	pheno.447.1.GATE	UKB GATE	Stricture of artery	2435	0.01	0.73	0.00	0.22	0.03	1038	396788
Locus 3	pheno.427.8.GATE	UKB GATE	Sinoatrial node dysfunction (Bradycardia)	2435	0.01	0.80	0.00	0.16	0.03	575	373492
Locus 3	pheno.446.GATE	UKB GATE	Polyarteritis nodosa and allied conditions	2435	0.01	0.80	0.00	0.15	0.03	1018	396788
Locus 3	pheno.415.21.GATE	UKB GATE	Primary pulmonary hypertension	2435	0.01	0.80	0.00	0.15	0.03	502	399568
Locus 3	pheno.418.1.GATE	UKB GATE	Precordial pain	2435	0.01	0.81	0.00	0.14	0.03	4507	373568
Locus 3	pheno.791.GATE	UKB GATE	Gangrene	2435	0.01	0.81	0.00	0.14	0.03	776	407806
Locus 3	pheno.401.22.GATE	UKB GATE	Hypertensive chronic kidney disease	2435	0.01	0.83	0.00	0.13	0.03	1583	315595
Locus 3	pheno.446.5.GATE	UKB GATE	Giant cell arteritis	2435	0.01	0.83	0.00	0.13	0.03	517	396788
Locus 3	pheno.420.3.GATE	UKB GATE	Endocarditis	2435	0.01	0.84	0.00	0.12	0.03	917	404327
Locus 3	pheno.427.6.GATE	UKB GATE	Premature beats	2435	0.01	0.84	0.00	0.12	0.03	746	373492
Locus 3	pheno.426.3.GATE	UKB GATE	Bundle branch block	2435	0.01	0.85	0.00	0.11	0.03	4663	373492
Locus 3	pheno.798.GATE	UKB GATE	Malaise and fatigue	2435	0.01	0.85	0.00	0.11	0.03	4197	404385
Locus 3	pheno.430.GATE	UKB GATE	Intracranial hemorrhage	2435	0.01	0.85	0.00	0.11	0.03	2393	395927
Locus 3	pheno.444.GATE	UKB GATE	Arterial embolism and thrombosis	2435	0.01	0.85	0.00	0.10	0.03	1099	396788
Locus 3	pheno.426.32.GATE	UKB GATE	Left bundle branch block	2435	0.01	0.87	0.00	0.09	0.03	2482	373492
Locus 3	pheno.433.21.GATE	UKB GATE	Cerebral artery occlusion, with cerebral infarction	2435	0.01	0.87	0.00	0.09	0.03	2063	395927
Locus 3	pheno.793.2.GATE	UKB GATE	Nonspecific abnormal findings on radiological and other examination of other intrathoracic organs (echocardiogram, etc)	2435	0.01	0.62	0.01	0.34	0.02	747	407656

Locus 3	pheno.426.31.GATE	UKB GATE	Right bundle branch block	2435	0.01	0.72	0.00	0.24	0.02	2150	373492
Locus 3	pheno.450.GATE	UKB GATE	Noninfectious disorders of lymphatic channels	2435	0.01	0.76	0.00	0.21	0.02	978	407604
Locus 3	pheno.401.2.GATE	UKB GATE	Hypertensive heart and/or renal disease	2435	0.01	0.79	0.00	0.17	0.02	2048	315595
Locus 3	pheno.402.GATE	UKB GATE	Elevated blood pressure reading without diagnosis of hypertension	2435	0.01	0.79	0.00	0.17	0.02	1793	315595
Locus 3	pheno.395.2.GATE	UKB GATE	Nonrheumatic aortic valve disorders	2435	0.01	0.82	0.00	0.15	0.02	3435	389441
Locus 3	pheno.395.1.GATE	UKB GATE	Nonrheumatic mitral valve disorders	2435	0.01	0.82	0.00	0.15	0.02	3088	389441
Locus 3	pheno.415.2.GATE	UKB GATE	Chronic pulmonary heart disease	2435	0.01	0.81	0.00	0.15	0.02	724	399568
Locus 3	pheno.451.2.GATE	UKB GATE	Phlebitis and thrombophlebitis of lower extremities	2435	0.01	0.83	0.00	0.13	0.02	4406	365238
Locus 3	pheno.429.GATE	UKB GATE	Ill-defined descriptions and complications of heart disease	2435	0.01	0.83	0.00	0.13	0.02	1028	399998
Locus 3	pheno.433.31.GATE	UKB GATE	Transient cerebral ischemia	2435	0.01	0.84	0.00	0.12	0.02	2559	395927
Locus 3	pheno.770.GATE	UKB GATE	Myalgia and myositis unspecified	2435	0.01	0.84	0.00	0.12	0.02	909	407673
Locus 3	pheno.425.1.GATE	UKB GATE	Primary/intrinsic cardiomyopathies	2435	0.01	0.86	0.00	0.11	0.02	1620	404327
Locus 3	pheno.771.GATE	UKB GATE	Musculoskeletal symptoms referable to limbs	2435	0.01	0.86	0.00	0.10	0.02	960	401991
Locus 3	pheno.411.9.GATE	UKB GATE	Other acute and subacute forms of ischemic heart disease	2435	0.01	0.87	0.00	0.09	0.02	1983	370814
Locus 3	pheno.395.6.GATE	UKB GATE	Heart valve replaced	2435	0.01	0.88	0.00	0.09	0.02	1891	389441
Locus 3	pheno.766.GATE	UKB GATE	Neuralgia, neuritis, and radiculitis NOS	2435	0.01	0.87	0.00	0.09	0.02	1563	403696
Locus 3	pheno.426.21.GATE	UKB GATE	First degree AV block	2435	0.01	0.87	0.00	0.09	0.02	1481	373492
Locus 3	pheno.451.GATE	UKB GATE	Phlebitis and thrombophlebitis	2435	0.01	0.89	0.00	0.08	0.02	4779	365238
Locus 3	pheno.761.GATE	UKB GATE	Cervicalgia	2435	0.01	0.88	0.00	0.08	0.02	2157	406425
Locus 3	pheno.440.GATE	UKB GATE	Atherosclerosis	2435	0.01	0.89	0.00	0.08	0.02	1781	396788
Locus 3	pheno.441.GATE	UKB GATE	Vascular insufficiency of intestine	2435	0.01	0.89	0.00	0.08	0.02	1429	396788
Locus 3	pheno.458.1.GATE	UKB GATE	Orthostatic hypotension	2435	0.01	0.90	0.00	0.07	0.02	1981	381440
Locus 3	pheno.781.GATE	UKB GATE	Symptoms involving nervous and musculoskeletal systems	2435	0.01	0.91	0.00	0.06	0.02	8903	399677
Locus 3	pheno.458.9.GATE	UKB GATE	Hypotension NOS	2435	0.01	0.92	0.00	0.05	0.02	6949	381440
Locus 3	pheno.433.8.GATE	UKB GATE	Late effects of cerebrovascular disease	2435	0.01	0.69	0.00	0.28	0.01	1640	395927
Locus 3	pheno.416.GATE	UKB GATE	Cardiomegaly	2435	0.01	0.79	0.00	0.18	0.01	3758	399568
Locus 3	pheno.433.3.GATE	UKB GATE	Cerebral ischemia	2435	0.01	0.83	0.00	0.15	0.01	5602	395927
Locus 3	pheno.790.6.GATE	UKB GATE	Other abnormal blood chemistry	2435	0.01	0.85	0.00	0.13	0.01	8303	400012
Locus 3	pheno.411.1.GATE	UKB GATE	Unstable angina (intermediate coronary syndrome)	2435	0.01	0.85	0.00	0.13	0.01	5505	370814
Locus 3	pheno.396.GATE	UKB GATE	Abnormal heart sounds	2435	0.01	0.85	0.00	0.12	0.01	11861	389441
Locus 3	pheno.788.GATE	UKB GATE	Syncope and collapse	2435	0.01	0.86	0.00	0.12	0.01	11041	397541
Locus 3	pheno.426.91.GATE	UKB GATE	Cardiac pacemaker in situ	2435	0.01	0.86	0.00	0.11	0.01	3082	373492
Locus 3	pheno.454.GATE	UKB GATE	Varicose veins	2435	0.01	0.87	0.00	0.10	0.01	13156	365238
Locus 3	pheno.454.1.GATE	UKB GATE	Varicose veins of lower extremity	2435	0.01	0.88	0.00	0.10	0.01	12611	365238
Locus 3	pheno.415.GATE	UKB GATE	Pulmonary heart disease	2435	0.01	0.87	0.00	0.10	0.01	5635	399568
Locus 3	pheno.783.GATE	UKB GATE	Fever of unknown origin	2435	0.01	0.88	0.00	0.10	0.01	4869	403713
Locus 3	pheno.426.9.GATE	UKB GATE	Cardiac pacemaker/device in situ	2435	0.01	0.88	0.00	0.09	0.01	3332	373492
Locus 3	pheno.764.GATE	UKB GATE	Sciatica	2435	0.01	0.89	0.00	0.09	0.01	2906	403696
Locus 3	pheno.411.3.GATE	UKB GATE	Angina pectoris	2435	0.01	0.90	0.00	0.08	0.01	20875	370814
Locus 3	pheno.415.1.GATE	UKB GATE	Acute pulmonary heart disease	2435	0.01	0.89	0.00	0.08	0.01	4940	399568
Locus 3	pheno.415.11.GATE	UKB GATE	Pulmonary embolism and infarction, acute	2435	0.01	0.89	0.00	0.08	0.01	4940	399568
Locus 3	pheno.426.2.GATE	UKB GATE	Atrioventricular [AV] block	2435	0.01	0.89	0.00	0.08	0.01	3012	373492
Locus 3	pheno.420.GATE	UKB GATE	Carditis	2435	0.01	0.89	0.00	0.08	0.01	2750	404327
Locus 3	pheno.760.GATE	UKB GATE	Back pain	2435	0.01	0.91	0.00	0.07	0.01	17230	391352
Locus 3	pheno.428.2.GATE	UKB GATE	Heart failure NOS	2435	0.01	0.91	0.00	0.07	0.01	7624	399998
Locus 3	pheno.458.GATE	UKB GATE	Hypotension	2435	0.01	0.92	0.00	0.06	0.01	8152	381440
Locus 3	pheno.773.GATE	UKB GATE	Pain in limb	2435	0.01	0.92	0.00	0.06	0.01	7405	401177
Locus 3	pheno.771.1.GATE	UKB GATE	Swelling of limb	2435	0.01	0.92	0.00	0.06	0.01	5688	401991
Locus 3	pheno.455.GATE	UKB GATE	Hemorrhoids	2435	0.01	0.92	0.00	0.05	0.01	26034	365238
Locus 3	pheno.459.GATE	UKB GATE	Other disorders of circulatory system	2435	0.01	0.93	0.00	0.05	0.01	21015	381440
Locus 3	pheno.459.9.GATE	UKB GATE	Circulatory disease NEC	2435	0.01	0.93	0.00	0.05	0.01	20772	381440
Locus 3	pheno.433.GATE	UKB GATE	Cerebrovascular disease	2435	0.01	0.93	0.00	0.05	0.01	11116	395927
Locus 3	pheno.411.2.GATE	UKB GATE	Myocardial infarction	2435	0.01	0.94	0.00	0.04	0.01	14781	370814
Locus 3	pheno.418.GATE	UKB GATE	Nonspecific chest pain	2435	0.02	0.95	0.00	0.03	0.01	32343	373568
Locus 3	pheno.785.GATE	UKB GATE	Abdominal pain	2435	0.02	0.96	0.00	0.02	0.01	46029	362553
Locus 3	pheno.401.GATE	UKB GATE	Hypertension	2435	0.00	0.00	0.02	0.98	0.00	92267	315595
Locus 3	pheno.401.1.GATE	UKB GATE	Essential hypertension	2435	0.00	0.00	0.02	0.98	0.00	91962	315595
Locus 3	pheno.411.8.GATE	UKB GATE	Other chronic ischemic heart disease, unspecified	2435	0.01	0.89	0.00	0.09	0.00	30853	370814
Locus 3	pheno.411.GATE	UKB GATE	Ischemic Heart Disease	2435	0.01	0.92	0.00	0.06	0.00	36962	370814
Locus 3	COVID19_HGI_A2_ALL_leave_23andme_20210107	HGI	Very severe respiratory confirmed covid vs. population	2317	0.01	0.89	0.00	0.08	0.02	5582	709010
Locus 3	COVID19_HGI_B1_ALL_eur_leave_ukbb_23andme_20210107	HGI	Hospitalized covid vs. not hospitalized covid (EUR)	2238	0.02	0.91	0.00	0.06	0.02	3159	7206
Locus 3	COVID19_HGI_B1_ALL_leave_UKBB_23andme_20210107	HGI	Hospitalized covid vs. not hospitalized covid	2125	0.02	0.92	0.00	0.04	0.02	3961	10538
Locus 3	COVID19_HGI_A2_ALL_eur_leave_23andme_20210107	HGI	Very severe respiratory confirmed covid vs. population (EUR)	2439	0.01	0.88	0.00	0.09	0.01	5101	1383241
Locus 3	COVID19_HGI_B1_ALL_eur_leave_23andme_20210107	HGI	Hospitalized covid vs. not hospitalized covid (EUR)	2238	0.02	0.89	0.00	0.08	0.01	4829	11816
Locus 3	COVID19_HGI_A2_ALL_eur_leave_ukbb_23andme_20210107	HGI	Very severe respiratory confirmed covid vs. population (EUR)	2449	0.01	0.89	0.00	0.08	0.01	4792	1054664
Locus 3	COVID19_HGI_B2_ALL_eur_leave_ukbb_23andme_20210107	HGI	Hospitalized covid vs. population (EUR)	2313	0.02	0.91	0.00	0.07	0.01	8316	1549095
Locus 3	COVID19_HGI_A2_ALL_leave_UKBB_23andme_20210107	HGI	Very severe respiratory confirmed covid vs. population	2320	0.01	0.90	0.00	0.07	0.01	5870	1155203
Locus 3	COVID19_HGI_B2_ALL_eur_leave_23andme_20210107	HGI	Hospitalized covid vs. population (EUR)	2313	0.02	0.92	0.00	0.06	0.01	9986	1877672
Locus 3	COVID19_HGI_B1_ALL_leave_23andme_20210107	HGI	Hospitalized covid vs. not hospitalized covid	2287	0.02	0.92	0.00	0.05	0.01	5773	15497
Locus 3	COVID19_HGI_B2_ALL_leave_23andme_20210107	HGI	Hospitalized covid vs. population	2322	0.02	0.93	0.00	0.04	0.01	12888	1295966
Locus 3	COVID19_HGI_B2_ALL_leave_UKBB_23andme_20210107	HGI	Hospitalized covid vs. population	2166	0.02	0.93	0.00	0.04	0.01	11829	1725210
Locus 3	COVID19_HGI_C2_ALL_leave_23andme_20210107	HGI	Covid vs. population	2398	0.02	0.95	0.00	0.03	0.01	36590	1668938
Locus 3	COVID19_HGI_C2_ALL_eur_leave_23andme_20210107	HGI	Covid vs. population (EUR)	2420	0.02	0.96	0.00	0.02	0.01	38984	1644784
Locus 3	COVID19_HGI_C2_ALL_leave_UKBB_23andme_20210107	HGI	Covid vs. population	2362	0.01	0.94	0.00	0.04	0.00	42557	1424707
Locus 3	COVID19_HGI_C2_ALL_eur_leave_ukbb_23andme_20210107	HGI	Covid vs. population (EUR)	2421	0.02	0.95	0.00	0.03	0.00	32494	1316207
Locus 3	ENSG00000113407.9 in Whole_Blood	GTEx cis-eQTL	ENSG00000113407.9 in Whole_Blood	2017	0.01	0.57	0.00	0.10	0.32	NA	369
Locus 3	ENSG00000272086.1 in Artery_Coronary	GTEx cis-eQTL	ENSG00000272086.1 in Artery_Coronary	2105	0.01	0.73	0.00	0.15	0.11	NA	152
Locus 3	ENSG00000113407.9 in Artery_Tibial	GTEx cis-eQTL	ENSG00000113407.9 in Artery_Tibial	2017	0.01	0.76	0.00	0.12	0.11	NA	388
Locus 3	ENSG00000056097.11 in Artery_Aorta	GTEx cis-eQTL	ENSG00000056097.11 in Artery_Aorta	2354	0.01	0.72	0.00	0.18	0.09	NA	267
Locus 3	ENSG00000250764.1 in Artery_Tibial	GTEx cis-eQTL	ENSG00000250764.1 in Artery_Tibial	1965	0.01	0.75	0.00	0.15	0.09	NA	388
Locus 3	ENSG00000056097.11 in Artery_Tibial	GTEx cis-eQTL	ENSG00000056097.11 in Artery_Tibial	2354	0.01	0.73	0.00	0.18	0.08	NA	388
Locus 3	ENSG00000056097.11 in Whole_Blood	GTEx cis-eQTL	ENSG00000056097.11 in Whole_Blood	2354	0.01	0.73	0.00	0.19	0.07	NA	369
Locus 3	ENSG00000113387.7 in Artery_Coronary	GTEx cis-eQTL	ENSG00000113387.7 in Artery_Coronary	2354	0.01	0.74	0.00	0.17	0.07	NA	152
Locus 3	ENSG00000113384.9 in Artery_Coronary	GTEx cis-eQTL	ENSG00000113384.9 in Artery_Coronary	2105	0.01	0.75	0.00	0.16	0.07	NA	152
Locus 3	ENSG00000250764.1 in Heart_Left_Ventricle	GTEx cis-eQTL	ENSG00000250764.1 in Heart_Left_Ventricle	1965	0.01	0.75	0.00	0.16	0.07	NA	272
Locus 3	ENSG00000150712.6 in Artery_Coronary	GTEx cis-eQTL	ENSG00000150712.6 in Artery_Coronary	2354	0.01	0.72	0.00	0.20	0.06	NA	152
Locus 3	ENSG00000113389.11 in Artery_Coronary	GTEx cis-eQTL	ENSG00000113389.11 in Artery_Coronary	2354	0.01	0.73	0.00	0.20	0.06	NA	152
Locus 3	ENSG00000113384.9 in Artery_Tibial	GTEx cis-eQTL	ENSG00000113384.9 in Artery_Tibial	2105	0.01	0.76	0.00	0.17	0.06	NA	388
Locus 3	ENSG00000113389.11 in Artery_Tibial	GTEx cis-eQTL	ENSG00000113389.11 in Artery_Tibial	2354	0.01	0.76	0.00	0.17	0.06	NA	388
Locus 3	ENSG00000240376.1 in Heart_Left_Ventricle	GTEx cis-eQTL	ENSG00000240376.1 in Heart_Left_Ventricle	2354	0.01	0.76	0.00	0.16	0.06	NA	272
Locus 3	ENSG00000113407.9 in Heart_Left_Ventricle	GTEx cis-eQTL	ENSG00000113407.9 in Heart_Left_Ventricle	2017	0.01	0.77	0.00	0.16	0.06	NA	272
Locus 3	ENSG00000056097.11 in Artery_Coronary	GTEx cis-eQTL	ENSG00000056097.11 in Artery_Coronary	2354	0.01	0.78	0.00	0.15	0.06	NA	152
Locus 3	ENSG00000250764.1 in Artery_Coronary	GTEx cis-eQTL	ENSG00000250764.1 in Artery_Coronary	1965	0.01	0.78	0.00	0.15	0.06	NA	152
Locus 3	ENSG00000113407.9 in Artery_Coronary	GTEx cis-eQTL	ENSG00000113407.9 in Artery_Coronary	2017	0.01	0.78	0.00	0.14	0.06	NA	152
Locus 3	ENSG00000113407.9 in Artery_Aorta	GTEx cis-eQTL	ENSG00000113407.9 in Artery_Aorta	2017	0.01	0.80	0.00	0.13	0.06	NA	267
Locus 3	ENSG00000243914.1 in Heart_Left_Ventricle	GTEx cis-eQTL	ENSG00000243914.1 in Heart_Left_Ventricle	1251	0.01	0.83	0.00	0.10	0.06	NA	272
Locus 3	ENSG00000150712.6 in Artery_Aorta	GTEx cis-eQTL	ENSG00000150712.6 in Artery_Aorta	2354	0.01	0.64	0.00	0.30	0.05	NA	267
Locus 3	ENSG00000249572.1 in Artery_Coronary	GTEx cis-eQTL	ENSG00000249572.1 in Artery_Coronary	2017	0.01	0.66	0.00	0.27	0.05	NA	152
Locus 3	ENSG00000240376.1 in Artery_Coronary	GTEx cis-eQTL	ENSG00000240376.1 in Artery_Coronary	2354	0.01	0.70	0.00	0.24	0.05	NA	152
Locus 3	ENSG00000250697.1 in Artery_Coronary	GTEx cis-eQTL	ENSG00000250697.1 in Artery_Coronary	2288	0.01	0.72	0.00	0.21	0.05	NA	152
Locus 3	ENSG00000113389.11 in Heart_Left_Ventricle	GTEx cis-eQTL	ENSG00000113389.11 in Heart_Left_Ventricle	2354	0.01	0.75	0.00	0.18	0.05	NA	272
Locus 3	ENSG00000113387.7 in Heart_Left_Ventricle	GTEx cis-eQTL	ENSG00000113387.7 in Heart_Left_Ventricle	2354	0.01	0.76	0.00	0.18	0.05	NA	272
Locus 3	ENSG00000113387.7 in Artery_Aorta	GTEx cis-eQTL	ENSG00000113387.7 in Artery_Aorta	2354	0.01	0.76	0.00	0.18	0.05	NA	267
Locus 3	ENSG00000240376.1 in Artery_Aorta	GTEx cis-eQTL	ENSG00000240376.1 in Artery_Aorta	2354	0.01	0.77	0.00	0.17	0.05	NA	267
Locus 3	ENSG00000249572.1 in Heart_Left_Ventricle	GTEx cis-eQTL	ENSG00000249572.1 in Heart_Left_Ventricle	2017	0.01	0.77	0.00	0.17	0.05	NA	272
Locus 3	ENSG00000113384.9 in Artery_Aorta	GTEx cis-eQTL	ENSG00000113384.9 in Artery_Aorta	2105	0.01	0.78	0.00	0.15	0.05	NA	267
Locus 3	ENSG00000113384.9 in Heart_Left_Ventricle	GTEx cis-eQTL	ENSG00000113384.9 in Heart_Left_Ventricle	2105	0.01	0.79	0.00	0.15	0.05	NA	272
Locus 3	ENSG00000113389.11 in Artery_Aorta	GTEx cis-eQTL	ENSG00000113389.11 in Artery_Aorta	2354	0.01	0.79	0.00	0.15	0.05	NA	267
Locus 3	ENSG00000250764.1 in Artery_Aorta	GTEx cis-eQTL	ENSG00000250764.1 in Artery_Aorta	1965	0.01	0.80	0.00	0.14	0.05	NA	267
Locus 3	ENSG00000272086.1 in Artery_Aorta	GTEx cis-eQTL	ENSG00000272086.1 in Artery_Aorta	2105	0.01	0.81	0.00	0.13	0.05	NA	267
Locus 3	ENSG00000272086.1 in Heart_Left_Ventricle	GTEx cis-eQTL	ENSG00000272086.1 in Heart_Left_Ventricle	2105	0.01	0.80	0.00	0.13	0.05	NA	272
Locus 3	ENSG00000250764.1 in Whole_Blood	GTEx cis-eQTL	ENSG00000250764.1 in Whole_Blood	1965	0.01	0.81	0.00	0.13	0.05	NA	369
Locus 3	ENSG00000113384.9 in Whole_Blood	GTEx cis-eQTL	ENSG00000113384.9 in Whole_Blood	2105	0.01	0.81	0.00	0.13	0.05	NA	369
Locus 3	ENSG00000150712.6 in Heart_Left_Ventricle	GTEx cis-eQTL	ENSG00000150712.6 in Heart_Left_Ventricle	2354	0.01	0.67	0.00	0.28	0.04	NA	272
Locus 3	ENSG00000113387.7 in Whole_Blood	GTEx cis-eQTL	ENSG00000113387.7 in Whole_Blood	2354	0.01	0.74	0.00	0.20	0.04	NA	369
Locus 3	ENSG00000249572.1 in Whole_Blood	GTEx cis-eQTL	ENSG00000249572.1 in Whole_Blood	2017	0.01	0.76	0.00	0.18	0.04	NA	369
Locus 3	ENSG00000240376.1 in Artery_Tibial	GTEx cis-eQTL	ENSG00000240376.1 in Artery_Tibial	2354	0.01	0.78	0.00	0.17	0.04	NA	388
Locus 3	ENSG00000150712.6 in Whole_Blood	GTEx cis-eQTL	ENSG00000150712.6 in Whole_Blood	2354	0.01	0.78	0.00	0.17	0.04	NA	369
Locus 3	ENSG00000056097.11 in Heart_Left_Ventricle	GTEx cis-eQTL	ENSG00000056097.11 in Heart_Left_Ventricle	2354	0.01	0.78	0.00	0.16	0.04	NA	272
Locus 3	ENSG00000272086.1 in Whole_Blood	GTEx cis-eQTL	ENSG00000272086.1 in Whole_Blood	2105	0.01	0.78	0.00	0.16	0.04	NA	369
Locus 3	ENSG00000230829.3 in Whole_Blood	GTEx cis-eQTL	ENSG00000230829.3 in Whole_Blood	2282	0.01	0.79	0.00	0.16	0.04	NA	369
Locus 3	ENSG00000240376.1 in Whole_Blood	GTEx cis-eQTL	ENSG00000240376.1 in Whole_Blood	2354	0.01	0.79	0.00	0.15	0.04	NA	369
Locus 3	ENSG00000113387.7 in Artery_Tibial	GTEx cis-eQTL	ENSG00000113387.7 in Artery_Tibial	2354	0.01	0.81	0.00	0.13	0.04	NA	388

Locus 3	ENSG00000272086.1 in Artery_Tibial	GTEx cis-eQTL	ENSG00000272086.1 in Artery_Tibial	2105	0.01	0.81	0.00	0.13	0.04	NA	388
Locus 3	ENSG00000250697.1 in Artery_Aorta	GTEx cis-eQTL	ENSG00000250697.1 in Artery_Aorta	2288	0.01	0.42	0.01	0.54	0.03	NA	267
Locus 3	ENSG00000150712.6 in Artery_Tibial	GTEx cis-eQTL	ENSG00000150712.6 in Artery_Tibial	2354	0.01	0.60	0.01	0.35	0.03	NA	388
Locus 3	ENSG00000249572.1 in Artery_Aorta	GTEx cis-eQTL	ENSG00000249572.1 in Artery_Aorta	2017	0.00	0.20	0.01	0.77	0.02	NA	267
Locus 3	ENSG00000133401.11 in Whole_Blood	GTEx cis-eQTL	ENSG00000133401.11 in Whole_Blood	761	0.77	0.05	0.16	0.01	0.01	NA	369
Locus 3	ENSG00000242110.3 in Artery_Tibial	GTEx cis-eQTL	ENSG00000242110.3 in Artery_Tibial	699	0.70	0.03	0.25	0.01	0.01	NA	388
Locus 3	ENSG00000151388.6 in Heart_Left_Ventricle	GTEx cis-eQTL	ENSG00000151388.6 in Heart_Left_Ventricle	1005	0.81	0.05	0.12	0.01	0.01	NA	272
Locus 3	ENSG00000133401.11 in Artery_Aorta	GTEx cis-eQTL	ENSG00000133401.11 in Artery_Aorta	761	0.84	0.05	0.09	0.01	0.01	NA	267
Locus 3	ENSG00000133401.11 in Heart_Left_Ventricle	GTEx cis-eQTL	ENSG00000133401.11 in Heart_Left_Ventricle	761	0.86	0.05	0.07	0.00	0.01	NA	272
Locus 3	ENSG00000082196.16 in Artery_Aorta	GTEx cis-eQTL	ENSG00000082196.16 in Artery_Aorta	640	0.83	0.03	0.13	0.00	0.01	NA	267
Locus 3	ENSG00000133401.11 in Artery_Coronary	GTEx cis-eQTL	ENSG00000133401.11 in Artery_Coronary	761	0.88	0.06	0.06	0.00	0.01	NA	152
Locus 3	ENSG00000113360.12 in Artery_Aorta	GTEx cis-eQTL	ENSG00000113360.12 in Artery_Aorta	606	0.90	0.05	0.05	0.00	0.01	NA	267
Locus 3	ENSG00000249572.1 in Artery_Tibial	GTEx cis-eQTL	ENSG00000249572.1 in Artery_Tibial	2017	0.00	0.02	0.02	0.96	0.00	NA	388
Locus 3	ENSG00000250697.1 in Artery_Tibial	GTEx cis-eQTL	ENSG00000250697.1 in Artery_Tibial	2288	0.00	0.05	0.02	0.93	0.00	NA	388
Locus 3	ENSG00000151388.6 in Artery_Coronary	GTEx cis-eQTL	ENSG00000151388.6 in Artery_Coronary	1005	0.87	0.05	0.07	0.00	0.00	NA	152
Locus 3	ENSG00000151388.6 in Artery_Aorta	GTEx cis-eQTL	ENSG00000151388.6 in Artery_Aorta	1005	0.87	0.05	0.07	0.00	0.00	NA	267
Locus 3	ENSG00000151388.6 in Artery_Tibial	GTEx cis-eQTL	ENSG00000151388.6 in Artery_Tibial	1005	0.87	0.05	0.07	0.00	0.00	NA	388
Locus 3	ENSG00000133401.11 in Artery_Tibial	GTEx cis-eQTL	ENSG00000133401.11 in Artery_Tibial	761	0.89	0.06	0.05	0.00	0.00	NA	388
Locus 3	ENSG00000242110.3 in Heart_Left_Ventricle	GTEx cis-eQTL	ENSG00000242110.3 in Heart_Left_Ventricle	699	0.89	0.04	0.07	0.00	0.00	NA	272
Locus 3	ENSG00000082213.13 in Artery_Coronary	GTEx cis-eQTL	ENSG00000082213.13 in Artery_Coronary	606	0.89	0.05	0.05	0.00	0.00	NA	152
Locus 3	ENSG00000113360.12 in Whole_Blood	GTEx cis-eQTL	ENSG00000113360.12 in Whole_Blood	606	0.90	0.05	0.05	0.00	0.00	NA	369
Locus 3	ENSG00000113360.12 in Artery_Coronary	GTEx cis-eQTL	ENSG00000113360.12 in Artery_Coronary	606	0.90	0.05	0.05	0.00	0.00	NA	152
Locus 3	ENSG00000082213.13 in Artery_Aorta	GTEx cis-eQTL	ENSG00000082213.13 in Artery_Aorta	606	0.90	0.05	0.04	0.00	0.00	NA	267
Locus 3	ENSG00000242110.3 in Artery_Aorta	GTEx cis-eQTL	ENSG00000242110.3 in Artery_Aorta	699	0.90	0.04	0.06	0.00	0.00	NA	267
Locus 3	ENSG00000082213.13 in Heart_Left_Ventricle	GTEx cis-eQTL	ENSG00000082213.13 in Heart_Left_Ventricle	606	0.91	0.05	0.04	0.00	0.00	NA	272
Locus 3	ENSG00000113360.12 in Heart_Left_Ventricle	GTEx cis-eQTL	ENSG00000113360.12 in Heart_Left_Ventricle	606	0.91	0.05	0.04	0.00	0.00	NA	272
Locus 3	ENSG00000242110.3 in Artery_Coronary	GTEx cis-eQTL	ENSG00000242110.3 in Artery_Coronary	699	0.91	0.04	0.05	0.00	0.00	NA	152
Locus 3	ENSG00000113360.12 in Artery_Tibial	GTEx cis-eQTL	ENSG00000113360.12 in Artery_Tibial	606	0.91	0.05	0.04	0.00	0.00	NA	388
Locus 3	ENSG00000082213.13 in Whole_Blood	GTEx cis-eQTL	ENSG00000082213.13 in Whole_Blood	606	0.91	0.05	0.04	0.00	0.00	NA	369
Locus 3	ENSG00000082213.13 in Artery_Tibial	GTEx cis-eQTL	ENSG00000082213.13 in Artery_Tibial	606	0.91	0.05	0.04	0.00	0.00	NA	388
Locus 3	ENSG00000082196.16 in Artery_Coronary	GTEx cis-eQTL	ENSG00000082196.16 in Artery_Coronary	640	0.91	0.03	0.05	0.00	0.00	NA	152
Locus 3	ENSG00000082196.16 in Heart_Left_Ventricle	GTEx cis-eQTL	ENSG00000082196.16 in Heart_Left_Ventricle	640	0.91	0.03	0.05	0.00	0.00	NA	272
Locus 3	ENSG00000242110.3 in Whole_Blood	GTEx cis-eQTL	ENSG00000242110.3 in Whole_Blood	699	0.91	0.04	0.04	0.00	0.00	NA	369
Locus 3	ENSG00000082196.16 in Artery_Tibial	GTEx cis-eQTL	ENSG00000082196.16 in Artery_Tibial	640	0.92	0.03	0.04	0.00	0.00	NA	388
Locus 3	ENSG00000082196.16 in Whole_Blood	GTEx cis-eQTL	ENSG00000082196.16 in Whole_Blood	640	0.92	0.03	0.04	0.00	0.00	NA	369
Locus 3	ENSG00000215156.5 in Artery_Aorta	GTEx cis-eQTL	ENSG00000215156.5 in Artery_Aorta	190	0.97	0.01	0.02	0.00	0.00	NA	267
Locus 3	ENSG00000215156.5 in Artery_Coronary	GTEx cis-eQTL	ENSG00000215156.5 in Artery_Coronary	190	0.98	0.01	0.01	0.00	0.00	NA	152
Locus 3	ENSG00000215156.5 in Artery_Tibial	GTEx cis-eQTL	ENSG00000215156.5 in Artery_Tibial	190	0.98	0.01	0.01	0.00	0.00	NA	388
Locus 3	ENSG00000215156.5 in Whole_Blood	GTEx cis-eQTL	ENSG00000215156.5 in Whole_Blood	190	0.98	0.01	0.01	0.00	0.00	NA	369
Locus 3	ENSG00000215158.5 in Artery_Coronary	GTEx cis-eQTL	ENSG00000215158.5 in Artery_Coronary	17	1.00	0.00	0.00	0.00	0.00	NA	152
Locus 3	ENSG00000215158.5 in Heart_Left_Ventricle	GTEx cis-eQTL	ENSG00000215158.5 in Heart_Left_Ventricle	17	1.00	0.00	0.00	0.00	0.00	NA	272
Locus 3	ENSG00000215158.5 in Artery_Tibial	GTEx cis-eQTL	ENSG00000215158.5 in Artery_Tibial	17	1.00	0.00	0.00	0.00	0.00	NA	388
Locus 3	ENSG00000215158.5 in Whole_Blood	GTEx cis-eQTL	ENSG00000215158.5 in Whole_Blood	17	1.00	0.00	0.00	0.00	0.00	NA	369
Locus 3	ENSG00000215158.5 in Artery_Aorta	GTEx cis-eQTL	ENSG00000215158.5 in Artery_Aorta	17	1.00	0.00	0.00	0.00	0.00	NA	267
Locus 3	finngen_R4_I9_CARDARR	FinnGen	Cardiac arrest	2330	0.01	0.67	0.00	0.27	0.05	772	97986
Locus 3	finngen_R4_I9_CARDMYOOTH	FinnGen	Cardiomyopathy, other and unspecified	2330	0.01	0.84	0.00	0.10	0.05	971	130879
Locus 3	finngen_R4_I9_OTHILLHEART	FinnGen	Other or ill-defined heart diseases	2330	0.01	0.84	0.00	0.10	0.05	583	130491
Locus 3	finngen_R4_I9_CARDMYOHYP	FinnGen	Cardiomyopathy, Hypertrophic obstructive	2330	0.01	0.74	0.00	0.20	0.04	251	130159
Locus 3	finngen_R4_I9_OTH	FinnGen	Other CVD	2330	0.01	0.85	0.00	0.09	0.04	1721	176899
Locus 3	finngen_R4_FG_OTH	FinnGen	Other CVD (FINNGEN)	2330	0.01	0.77	0.00	0.18	0.03	1398	98612
Locus 3	finngen_R4_FG_CARDMYO	FinnGen	Cardiomyopathy (excluding other)	2330	0.01	0.84	0.00	0.12	0.03	1730	134693
Locus 3	finngen_R4_I9_MYOCARD	FinnGen	Myocarditis	2330	0.01	0.84	0.00	0.12	0.03	626	97840
Locus 3	finngen_R4_I9_CARDMPRI	FinnGen	Cardiomyopathies, Primary/intrinsic	2330	0.01	0.84	0.00	0.11	0.03	1678	131586
Locus 3	finngen_R4_I9_K_CARDIAC	FinnGen	Death due to cardiac causes	2330	0.01	0.86	0.00	0.09	0.03	6314	176899
Locus 3	finngen_R4_I9_NONISCHCARDMYOP_STRICT	FinnGen	Nonischemic cardiomyopathy	2330	0.01	0.48	0.01	0.49	0.02	653	151314
Locus 3	finngen_R4_I9_AF_REIMB	FinnGen	Atrial fibrillation and flutter with reimbursement	2330	0.01	0.85	0.00	0.12	0.02	8171	105385
Locus 3	finngen_R4_I9_CARDMYO	FinnGen	Cardiomyopathy	2330	0.01	0.84	0.00	0.12	0.02	2342	132250
Locus 3	finngen_R4_I9_CABG	FinnGen	Coronary artery bypass grafting	2330	0.02	0.89	0.00	0.07	0.02	4449	171377
Locus 3	finngen_R4_I9_AF	FinnGen	Atrial fibrillation and flutter	2330	0.02	0.90	0.00	0.06	0.02	17325	114539
Locus 3	finngen_R4_I9_HEARTFAIL	FinnGen	Heart failure,strict	2330	0.01	0.55	0.01	0.43	0.01	9576	168862
Locus 3	finngen_R4_I9_HEARTFAIL_ALLCAUSE	FinnGen	All-cause Heart Failure	2330	0.01	0.56	0.01	0.42	0.01	17387	176445
Locus 3	finngen_R4_I9_MI_STRICT	FinnGen	Myocardial infarction, strict	2330	0.01	0.74	0.00	0.23	0.01	9145	161766
Locus 3	finngen_R4_I9_ANGIO	FinnGen	Coronary angiopasty	2330	0.01	0.75	0.00	0.23	0.01	6699	173627
Locus 3	finngen_R4_I9_ANGINA	FinnGen	Angina pectoris	2330	0.01	0.77	0.00	0.21	0.01	14712	167333
Locus 3	finngen_R4_I9_MI	FinnGen	Myocardial infarction	2330	0.01	0.77	0.00	0.20	0.01	10003	162624
Locus 3	finngen_R4_I9_CHD	FinnGen	Major coronary heart disease event	2330	0.01	0.85	0.00	0.13	0.01	16631	176899
Locus 3	finngen_R4_I9_CHD_NOREV	FinnGen	Major coronary heart disease event excluding revascularizations	2330	0.01	0.85	0.00	0.12	0.01	13809	173972
Locus 3	finngen_R4_I9_ISCHHEART	FinnGen	Ischemic heart diseases	2330	0.02	0.87	0.00	0.11	0.01	24278	176899
Locus 3	finngen_R4_I9_CVD_HARD	FinnGen	Hard cardiovascular diseases	2330	0.02	0.88	0.00	0.10	0.01	22860	176899
Locus 3	finngen_R4_I9_CORATHER	FinnGen	Coronary atherosclerosis	2330	0.02	0.89	0.00	0.08	0.01	18295	170916
Locus 3	finngen_R4_I9_HYPTENSHR	FinnGen	Hypertensive heart and/or renal disease	2330	0.02	0.89	0.00	0.08	0.01	3674	136997
Locus 3	finngen_R4_I9_HYPTENSHD	FinnGen	Hypertensive Heart Disease	2330	0.02	0.89	0.00	0.08	0.01	3252	136575
Locus 3	finngen_R4_FG_CVD	FinnGen	Cardiovascular diseases (excluding rheumatic etc)	2330	0.02	0.91	0.00	0.07	0.01	79685	176899
Locus 3	finngen_R4_I9_NONISCHCARDMYOP	FinnGen	Non-ischemic cardiomyopathy	2330	0.02	0.91	0.00	0.06	0.01	8300	151533
Locus 3	finngen_R4_I9_CVD	FinnGen	Cardiovascular diseases	2330	0.02	0.94	0.00	0.04	0.01	86957	176899
Locus 3	finngen_R4_FG_OTHHEART	FinnGen	Other heart diseases	2330	0.02	0.94	0.00	0.04	0.01	43936	141150
Locus 3	finngen_R4_I9_OTHHEART	FinnGen	Other heart diseases	2330	0.02	0.95	0.00	0.03	0.01	46991	176899
Locus 3	finngen_R4_I9_HYPERTENSION	FinnGen	Hypertensive diseases	2330	0.00	0.00	0.02	0.98	0.00	43576	176899
Locus 3	finngen_R4_I9_HYPTENSESS	FinnGen	Hypertension, essential	2330	0.00	0.01	0.02	0.97	0.00	33229	166552
Locus 3	finngen_R4_I9_HEARTFAIL_NS	FinnGen	Heart failure, not strict	2330	0.01	0.50	0.01	0.48	0.00	17613	176899
